# Viral entry defines the hepatitis E virus species barrier in murine hepatocytes

**DOI:** 10.1080/22221751.2026.2706321

**Published:** 2026-07-27

**Authors:** Nicola Frericks, Olinda Pinto Veiga, Leyla Sirkinti, Hoang Duy Nguyen, Tina Sliwinski, Hongbo Guo, Yannick Brüggemann, Thomas Burkard, Rainer G. Ulrich, Richard J.P. Brown, Tran Tuoc, Huu Phuc Nguyen, Wenshi Wang, Daniel Todt, Volker Kinast, Eike Steinmann

**Affiliations:** a Department of Molecular and Medical Virology, Ruhr University Bochum, Bochum, Germany; b Hepatitis E Virus Research Hub (HepE-Hub), Bochum, Germany; c Institute for Medical Microbiology and Virology, Carl von Ossietzky University Oldenburg, Oldenburg, Germany; d Department of Translational and Computational Infection Research (TRACiR), Ruhr University Bochum, Bochum, Germany; e Department of Human Genetics, Ruhr University Bochum, Bochum, Germany; f Department of Pathogen Biology and Immunology, Jiangsu Key Laboratory of Immunity and Metabolism, Jiangsu International Laboratory of Immunity and Metabolism, Xuzhou Medical University, Xuzhou, People’s Republic of China; g Institute of Novel and Emerging Infectious Diseases, Friedrich-Loeffler-Institut, Greifswald-Insel Riems, Germany; h European Virus Bioinformatics Center (EVBC), Jena, Germany

**Keywords:** Hepatitis E virus, zoonosis, species tropism, host factors, innate immunity

## Abstract

The *Paslahepevirus balayani* hepatitis E virus (HEV) and the distantly related *Rocahepevirus ratti* rat HEV pose a risk for zoonotic transmission to humans. Despite the broad host range, including its ability to infect certain rodent species, infections of animals of the genus *Mus* within the subfamily Murinae are rarely documented. Further, experimental infection of non-humanized mice has proven challenging. To dissect the molecular mechanisms underlying species barriers to HEV infection, this study aimed to investigate the virus replication cycle and immune-related determinants responsible for restricted HEV infection in murine hepatocytes. Murine hepatic cell lines supported moderate levels of zoonotic HEV replication and infectious virion production upon transfection of *in vitro* transcribed viral RNA. Notably, viral replication was not restricted by innate immune responses or presence of dominant restriction factors but was limited by absence of host-specific dependency factors in murine cell lines. While successful attachment to murine hepatic cells and recombinant capsid protein cleavage was observable, viral particle disassembly was not observed in murine hepatocytes, correlating with murine cell lines and primary murine hepatocytes being refractory to HEV infection. In summary, the murine barrier to HEV infection is defined at the viral entry stage, specifically by a block post attachment and before viral replication is initiated. These findings shed new light on the fundamental role of viral entry mechanisms in defining HEV species tropism.

## Introduction

Viral hepatitis is a leading cause of death by infectious agents globally, ranking second only to COVID-19, with the number of deaths rising in recent years (WHO). Infection with hepatitis E virus (HEV) is the most common cause of infectious acute hepatitis worldwide and represents a major global health problem [1]. Beyond chronic infections in immunocompromised patients, HEV infection of pregnant women is associated with a mortality rate up to 30% and heightened risk for adverse pregnancy outcomes [1]. The frequent spillover events from animal reservoirs highlight the ongoing risk to human health caused by zoonotic transmission of HEV [2].

HEV is a quasi-enveloped virus with a positive-sense single-stranded RNA genome classified in the *Hepeviridae* family. To date, eight HEV genotypes of the species *Paslahepevirus balayani* have been described. Infections with genotypes 1, 2, 3, 4 and 7 (HEV-1, −2, −3, −4, −7) have been detected in humans [3]. HEV-1 and HEV-2 spread through fecal-oral transmission exclusively within the human population, associated with inadequate sanitation and contaminated water sources in underdeveloped areas [4]. In contrast, infections with HEV-3, HEV-4 and HEV-7 in humans are mainly associated with the consumption of inadequately heated animal products and are therefore considered zoonotic [5–7]. HEV-3 is found in a variety of animal species, but zoonotic transmission most frequently originates from swine, wild boar, deer and rabbit [6,8–12]. Recent findings described the transmission of the distantly related rat HEV from the species *Rocahepevirus ratti* to humans [13–16], highlighting the importance of understanding molecular mechanisms of HEV cross-species transmission.

The establishment of a variety of *in vitro* systems has enabled the investigation of the molecular biology of HEV-3, HEV-4 and rat HEV infection in human, rat and more recently also in porcine hepatocytes [17–19]. Similarly, animal models have been developed using non-human primates, swine, rabbits and various rodent species [20]. While experimental infection of human liver chimeric mice has become a well-established HEV animal model [21], infection of non-humanized mice has proven challenging, with inconsistent results across studies. While some studies reported that certain mice strains are susceptible to HEV-4 infection, other groups have failed to reproduce these findings, even when using multiple HEV and various mouse strains, both immunocompetent and immunocompromised [22–27]. The inconsistency, together with the rarely documented natural infections in rodents of the genus *Mus* [28,29], prompted us to investigate the genetic and immune-related determinants that restrict HEV infection in this host. Here, we combine murine hepatocyte cultures with state-of-the-art approaches to study HEV replication and provide evidence that the initial barrier to HEV infection in murine cells is defined by a lack of susceptibility.

## Materials and methods

See online supplementary materials.

## Results

### Primary murine hepatocytes are refractory to HEV infection

**Figure 1.**
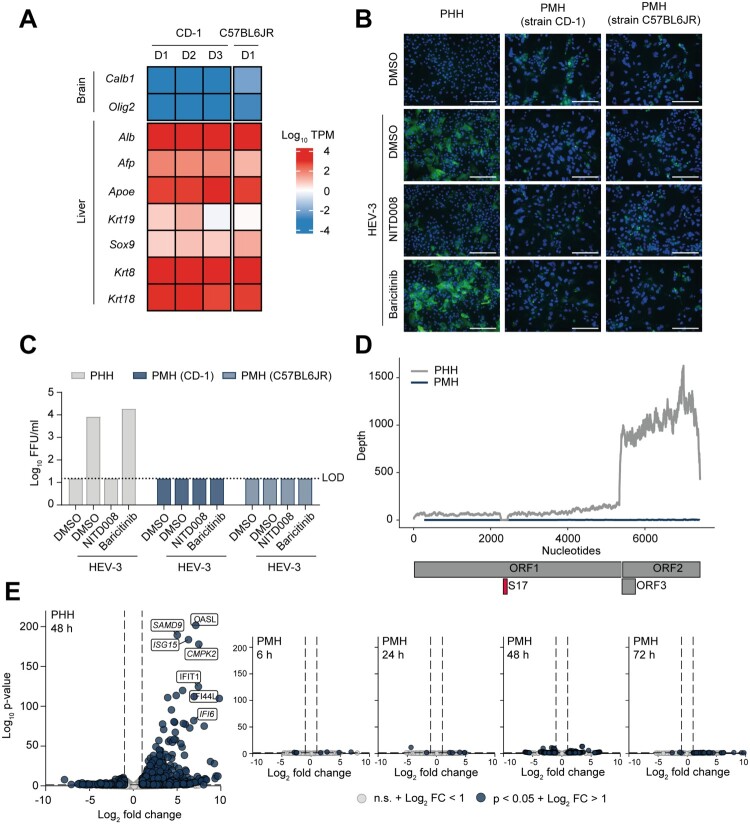
Primary murine hepatocytes are refractory to HEV-3 infection. (A) Heat maps of brain and liver tissue specific marker gene expression in primary murine hepatocytes. Transcripts per million (TPM) values from in total 4 individual donor mice of the CD-1 and C57BL6JR strain. (B) Representative immunofluorescence images stained for HEV-3 ORF2-encoded capsid protein in primary human hepatocytes (PHH, 1 donor) and primary mouse hepatocytes (PMH, 3 donors strain CD-1, 1 donor strain C57BL6JR) at 3 days post HEV-3 inoculation. Primary cells were either (pre-)treated with baricitinib [2 µM], NITD008 [0.5 µM] or with DMSO as solvent control. ORF2-encoded capsid protein = green; DAPI = blue; scale bar = 200 µm. (C) Progeny virus was harvested from lysates of primary cells at 3 days post HEV-3 inoculation and quantified via focus forming unit (FFU) assay on HepG2/C3A cells. Dashed line represents the limit of detection (LOD) with non-detectable samples that were set to LOD. Data presented derives from 1 donor each. (D) Depth of reads mapping to the HEV-3 Kernow-C1/p6 reference genome (GenBank accession no. JQ679013) in total RNA extracted from HEV-3 inoculated PHH (data was published previously [30]) and PMH (2 donors strain CD-1). Reads that mapped for the S17 insertion within the viral genome were excluded. The genome organization of HEV-3 Kernow-C1/p6 is shown below. (E) Volcano plots visualizing differentially expressed genes induced in HEV-3 inoculated and untreated PHH (3 donors, previously published in [31]) and PMH (2 donors, strain CD-1) at indicated time points.

To investigate the species-specific ability of hepatocytes to support HEV infection, we compared primary mouse hepatocytes (PMH) with primary human hepatocytes (PHH), the latter of which have been intensively characterized in previous studies [30,31]. Given the reported variability in HEV permissiveness among different mouse strains, we used commercially available PMH from two different mouse strains (CD-1 and C57BL6JR). RNA of four donor mice was subjected to total RNA sequencing (RNA-seq) to confirm expression of liver-specific marker genes (Figure 1(A)). Following this, both PMH and PHH were inoculated with infectious, cell culture-derived HEV-3 (HEVcc, Kernow-C1/p6). Given the high immunoreactivity of primary cells, we additionally performed (pre-)treatment with the Janus kinase (JAK) inhibitor baricitinib to preclude potential viral restriction by innate immune responses. Immunofluorescence staining for open reading frame (ORF) 2-encoded capsid protein expression upon inoculation with infectious HEVcc, showed robust viral protein expression in PHH, which was reduced by administration of the adenosine analogue inhibitor NITD008 (Figure 1(B)). In contrast, capsid protein-specific immunofluorescence staining remained undetectable in PMH regardless of baricitinib treatment. To preclude the possibility of insufficient viral protein levels for detection via this method, we additionally lysed the cells to harvest potential infectious particles and amplify the low levels by titration of the cell lysates on highly permissive HepG2/C3A cells. Consistent with the immunofluorescence staining results, infectious particles were successfully recovered from PHH but not from PMH lysates (Figure 1(C)). To further monitor the potential presence of low-level HEV replication in PMH, we applied RNA-seq, as the HEV genome mimics host mRNA, possessing a poly(A)tail. Mapping of reads to the HEV genome demonstrated a markedly lower abundance of HEV RNA in samples collected from PMH compared to PHH (Figure 1(D)), suggesting no establishment of productive HEV infection. Transcriptomic analyses of the host response further uncovered minimal differentially expressed genes (DEGs) between uninfected PMH and PMH inoculated with infectious HEVcc over time (Figure 1(E)), although high immunoreactivity upon stimulation with murine interferon-α (IFN-α) was confirmed (Figure S1). Notably, none of the DEGs in the murine HEV-inoculated condition were classified as antiviral genes, which are strongly deregulated in HEV-infected PHH (Figure 1(E)). Together, our *ex vivo* data suggest that PMH are refractory to HEV infection.

### Murine hepatic cell lines support HEV-3 replication

**Figure 2.**
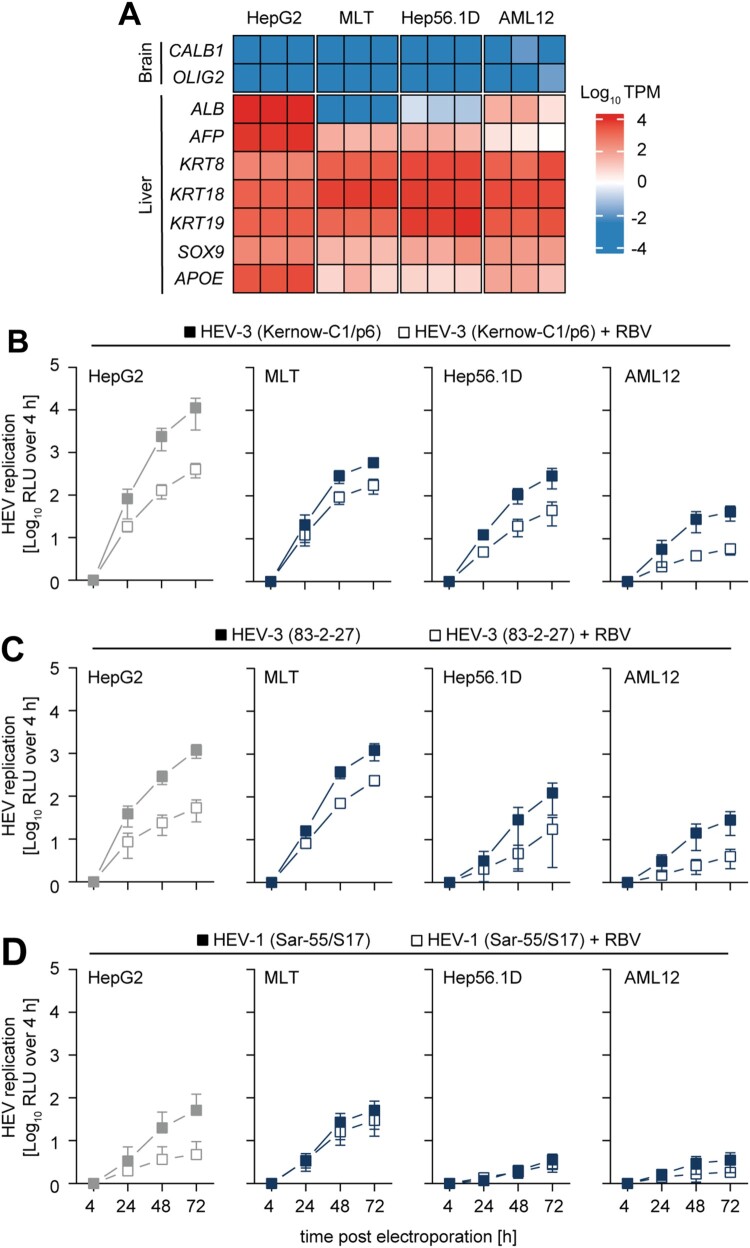
Murine liver cell lines support HEV-3 replication. (A) Heat maps of brain and liver tissue specific marker gene expression in human hepatoma HepG2 cells as well as murine hepatic cell lines MLT, Hep56.1D and AML12. Transcripts per million (TPM) values from three individual passages of cells are shown. (B, C, D) Replication fitness of HEV-3 (Kernow-C1/p6 and 83-2-27) and HEV-1 (Sar-55/S17) subgenomic replicons in human and murine liver cell lines with and without Ribavirin (RBV, 50 µM) treatment. Replication was measured at indicated time points in correlation to Gaussia luciferase activity. The presented data are relative light units (RLU) normalized to 4 h post electroporation. Mean values ± SD from n = 4 experiments are displayed.

Next, we assessed the HEV replication cycle in a panel of murine hepatic cell lines to identify the block preventing successful HEV infection. To have a broad spectrum of genetic background, we selected three different murine cell lines which were originally isolated from both healthy and tumoral livers of different mouse strains. The Hep56.1D cell line was isolated from a liver tumor of a C57BL/6J mouse [32]. The stable Mouse Liver Tumor (MLT) cell line was created by lentiviral gene transfer of oncogenic transposon plasmids to C57BL/J6 OlaHsd mice and further modified to express high levels of human miR-122 to support hepatitis C virus (HCV) infection [33]. The non-tumorigenic AML12 cell line was established from hepatocytes of a transgenic CD-1 mouse overexpressing the transforming growth factor alpha [34]. All three murine hepatic cell lines, which were previously used to study species barriers to hepatitis B virus (HBV) and hepatitis D virus (HDV) as well as HCV infection [33,35–38], show distinct expression levels of liver-associated mRNAs, albeit with differences to the human hepatocarcinoma cell line HepG2 (Figure 2(A)), which serves as the reference cell line to study HEV replication [39]. Notably, albumin expression was markedly lower in murine MLT and Hep56.1D cells. All three murine cell lines also show characteristic hepatocellular carcinoma marker gene expression of keratin 8 and 18 as well as expression of keratin 19, a marker of cholangiocarcinoma cells. Next, we focused on viral replication efficacy in murine hepatic cell lines by transfecting *in vitro* transcribed subgenomic RNA of the HEV-3 strain Kernow-C1/p6. Since a *Gaussia* luciferase reporter partially replaced sequences of the ORF2-encoded viral capsid protein in the replicon system, viral replication can be assessed independently from the viral entry as well as the efficacy of viral particle production, by correlation to the amounts of secreted *Gaussia* luciferase. Upon transfection of viral replicon RNA, murine hepatic cells supported HEV-3 replication with a 2–3 x log_10_ fold increase over time, with replication levels measured in HepG2 cells increasing more than 4 x log_10_ fold (Figure 2(B)). The infectious cDNA clone of the Kernow-C1/p6 strain originates from a clinical isolate of a chronically infected patient that was adapted to cell culture [40,41]. During the adaptation process, an insertion of the human S17 ribosomal protein sequence in the hypervariable region of the viral genome was selected and shown to confer significant growth advantages [40,41]. To exclude a strain-specific phenotype, we further investigated replication capacity of a subgenomic replicon based on the wild boar-derived HEV-3 strain 83-2-27 [42]. Similarly, replication of the HEV-3 strain 83-2-27 was detectable in murine hepatic cells as well as in HepG2 cells (Figure 2(C)). Interestingly, while for the Kernow-C1/p6 strain species-specific differences in replication efficiency between human and murine hepatocytes were observed, the 83-2-27 replicon replicated to similar levels in MLT cells when compared to HepG2 cells. While HEV-3 is classified as a zoonotic genotype, typically transmitted from animals to humans, HEV-1 circulates exclusively within the human population. Upon transfection of *in vitro* transcribed HEV-1 subgenomic replicon RNA based on the Sar55 strain, we detected relative light units counts, slightly higher than the replication inhibitor treated control (Figure 2(D)), indicating low levels of viral replication. However, the reason for the low replication levels remains uncertain. It could be attributed to the human-specific tropism of this genotype or the generally low *in vitro* replication fitness of this molecular clone, as also suggested by the moderate replication levels observed in HepG2 cells. In summary, murine hepatic cell lines support HEV-3 viral RNA replication albeit to a lower extent than the human hepatoblastoma cell line HepG2.

### Infectious HEV-3 particles assemble in murine hepatic cell lines

**Figure 3.**
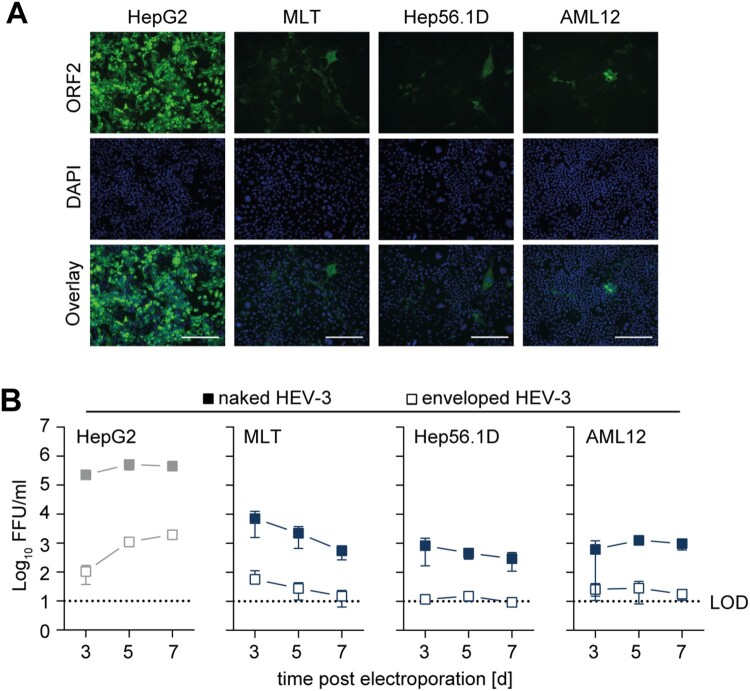
Infectious HEV-3 particles can be harvested from murine hepatic cells. (A) Representative immunofluorescence images (n = 3) of ORF2 capsid protein expression at 3 days post electroporation of *in vitro* transcribed full-length HEV-3 RNA. ORF2-encoded capsid protein = green; DAPI = blue; scale bar = 200 µm. (B) Viral titers of infectious HEV-3 particles harvested from human and murine cell lines at indicated time points after electroporation of *in vitro* transcribed full-length viral RNA. Naked virions were harvested from cell lysates whereas enveloped virions were collected from supernatant. Infectivity of harvested viruses were quantified via focus forming unit (FFU) assay on HepG2/C3A cells. Mean values ± SD from n = 3 experiments are shown. Non-detectable values for viral titres in independent experiments were set to the limit of detection (LOD).

In the next step, we investigated the capacity of murine hepatic cells to support assembly and release of infectious viral particles upon transfection of *in vitro* transcribed, full-length HEV-3 viral RNA. In line with moderate capsid protein expression being detectable after transfection (Figure 3(A)), robust production of infectious, naked HEV particles was confirmed in cell lysates (Figure 3(B)). Notably, while intracellular amounts of infectious particles increased over time in HepG2 cells, a decrease was observed in all three murine cell lines beyond 3 days post transfection. Consistent with this, HEV replication in HepG2 cells continued to increase beyond 72 h, whereas it plateaued at 72 h in murine cells (Figure S2). Moreover, the amount of enveloped viral particles secreted into the supernatant of murine hepatic cells were just above or at the limit of detection. Taken together, our data demonstrate that infectious HEV particles assemble and are released from murine hepatic cell lines, albeit with low efficiency.

### HEV-3 replication is highly sensitive to interferon-induced immune responses in murine hepatic cell lines

**Figure 4.**
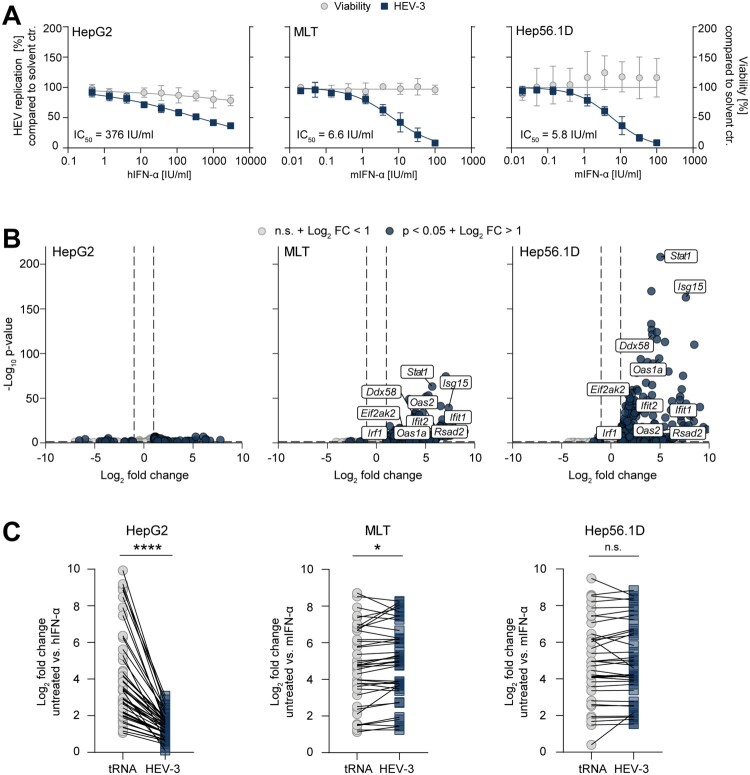
HEV-3 exhibits increased sensitivity against the murine interferon-stimulated response. (A) Dose-dependent antiviral activity of human and murine interferon-α (IFN-α) against HEV-3 replication. HEV replication was normalized to cells treated with the solvent control (ctr.). Normalized replication levels [%] were used for linear regression to determine half-maximal inhibitory concentration (IC_50_). Mean values ± SD are shown for n = 3 experiments. (B) Comparison of host transcriptional responses between untreated and IFN-α treated [100 IU/mL] HEV-transfected human and murine cells. N.s., not significant with *p*-value >0.05; *p* < 0.05 = *; *p* < 0.0001 = ****; FC, fold change (C) Comparison of the mean log_2_ fold change of n = 47 (human) and 38 (murine) selected IFN-stimulated genes (ISGs) of HEV-3 or tRNA transfected cells upon IFN-α treatment [100 IU/mL] compared to untreated. Statistical significance was calculated using two-tailed, paired t-test, *p* < 0.05 = *, *p* < 0.001 = ***. Presented RNA sequencing data derive from n = 3 experiments.

Genetic diversity among host species can lead to variations in innate immune response pathways or the inability of viral proteins to interact with homologous proteins of these pathways for immune evasion, resulting in restricted cross-species transmission. Thus, we first examined the immune reactivity of human and murine liver cell lines to non-HEV-specific stimuli. Both poly (I:C), a synthetic analog of double-stranded RNA that mimics viral genetic material, and species-specific IFN-α induced comparable antiviral responses in human and murine cells as evidenced by similarly elevated levels of ISG15/Isg15 and MX1/Mx1 mRNA (Figure S3). Based on this, we assessed if HEV infection is restricted by a murine innate immune response. Indeed, when we tested the antiviral effect of IFN-α on HEV replication, we observed an increased sensitivity to species-specific IFN-α treatment in murine cells compared to human cells, as demonstrated by 60-fold lower inhibitory concentration 50 (IC_50_) values (Figure 4(A)). In line with this, transcriptomic analyses of murine and human cells transfected with full-length HEV-3 RNA revealed a stronger induction of IFN-stimulated gene (ISG) expression in murine hepatic cells upon species-specific IFN-α treatment compared to their untreated control (Figure 4(B)). Previous literature provides evidence that HEV can counteract innate immune responses and thereby lower ISG expression levels [43]. In accordance with these studies, we observed milder induction of ISG expression upon IFN-α treatment in the presence of HEV-3 replication in human cells compared to cells that were transfected with human tRNA and treated with IFN-α as a control (Figures 4(C), S4). In contrast, induction of ISG expression was at similar levels upon murine IFN-α treatment between murine cells transfected with HEV-3 RNA or human tRNA control. In summary, HEV exhibits increased sensitivity to IFN-regulated immune responses in murine cells, likely due to its impaired ability to dampen IFN signalling in murine cells.

### Absence of a strong innate immune induction upon HEV-3 replication in murine hepatic cell lines

**Figure 5.**
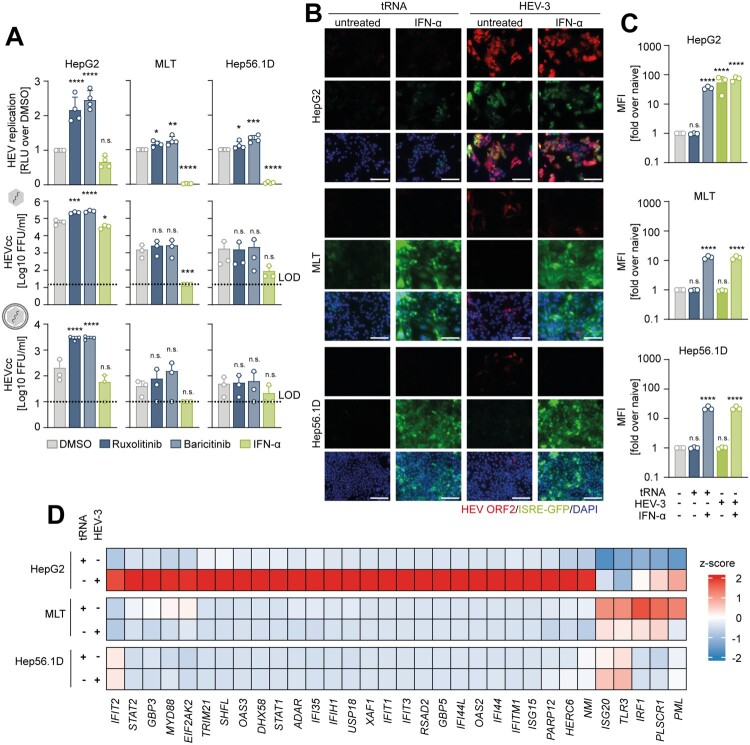
Absence of a strong innate immune response to HEC-3 infection in murine hepatic cells. (A) Impact of JAK inhibitors ruxolitinib and baricitinib [2 µM] as well as species-specific IFN-α treatment [100 IU/ml] on HEV-3 replication (upper), naked HEV-3 (middle) and enveloped HEV-3 (lower) particle production in HepG2, MLT and Hep56.1D cells at 72 h post transfection of sub genomic or infectious full-length RNA. The presented replication data are relative light units (RLU) normalized to the solvent control treated cells. Infectivity of harvested viruses were quantified via focus forming unit (FFU) assay on HepG2/C3A cells. Non-detectable values for viral titres were set to the limit of detection (LOD). Mean and individual values + SD from n = 3-4 experiments are shown. Statistical significance was calculated using an ordinary one-way ANOVA with Dunnett multiple comparison correction. Data of the virus production were log transformed prior to statistical analyses. N.s., not significant with *p*-value >0.05; * = *p*-value <0.05; ** = *p*-value <0.01; *** = *p*-value <0.001; **** = *p*-value <0.0001. (B, C) ISRE-promoter activity in HEV-3 transfected human and murine hepatic cell lines. Cells stably expressing a GFP under the ISRE promoter were transfected with *in vitro* transcribed full-length HEV-3 RNA or tRNA as a control. At 3 days post transfection, cells were fixed and stained for ORF2 capsid protein expression. As a positive control for promoter activity, cells were treated with 100 IU/mL IFN-α 16 h prior to fixation. (B) Representative immunofluorescence images of n = 3 experiments. ISRE-GFP = green; ORF2 capsid protein = red; DAPI = blue; scale bar = 100 µm. (C) Mean fluorescence intensity of expressed GFP measured via flow cytometry. Mean and individual values + SD from n = 3 experiments are shown. Statistical significance was calculated on log transformed data using an ordinary one-way ANOVA with Dunnett multiple comparison correction. N.s., not significant with *p*-value >0.05; * = *p*-value <0.05; **** = *p*-value <0.0001. (D) Heat map visualizing z-scores of selected genes clustered under the term “interferon stimulated genes” at 2 days post transfection of tRNA or HEV-3 full-length RNA. Presented RNA sequencing data originates from n = 3 experiments.

Based on these findings, we hypothesized that the murine species barrier to HEV infection is partly determined by innate immune-dependent antiviral restriction. We previously demonstrated that blocking JAK-dependent IFN signalling by baricitinib treatment facilitates HEV infection in human hepatocytes, correlating with reduced ISG expression in PHH [30]. To build on these findings, we investigated the impact of baricitinib treatment on HEV replication in murine cells compared to human cells. In line with our previous observations, we observed increased HEV replication and viral progeny production and release, correlating with reduced ISG expression upon baricitinib treatment of full-length and subgenomic HEV-3 transfected HepG2 cells (Figure 5(A) and S5). Notably, HEV-3 replication and virus production were inhibited by IFN-α treatment in murine cells, but a proviral effect of baricitinib treatment on viral replication was mild or not observed for viral particle production, despite confirmed activity of baricitinib in murine cells through the inhibition of IFN-α treatment-induced ISG expression (Figures 5(A), S5). Furthermore, IFN-stimulated response element (ISRE) reporter assays confirmed strong ISRE-dependent gene expression upon HEV-3 replication in human cells but only minor to absent expression in murine hepatic cell lines (Figures 5(B,C) and S6). Transcriptomic analyses further showed very mild ISG mRNA induction upon HEV-3 or tRNA transfection in murine cells, while robust induction was observed upon HEV replication in HepG2 cells (Figure 5(D)). Lastly, we investigated the impact of mitochondrial antiviral signalling protein (MAVS) knockout on HEV-3 replication in murine hepatocytes. MAVS is a key adaptor protein involved in downstream signalling after retinoic acid inducible gene I (RIG-I) like pattern recognition receptor activation, resulting in the production of IFNs and downstream induction of antiviral effector proteins. MLT cells from a MAVS knockout mouse were previously generated [33] and replication of both HEV-3 and HCV was assessed. HCV served as a control as it is known to stimulate and to be highly sensitive to murine innate immune responses [44]. In line with previous observations [33], MAVS knockout boosted HCV replication in MLT cells (Figure S7). In contrast, HEV-3 replication was detectable at similar levels in MLT cells with or without MAVS knockout. Collectively, our results indicate the absence of a strong innate immune response upon HEV-3 replication and progeny virus production in murine cell lines.

### Efficient HEV-3 replication in interspecies heterokaryons

**Figure 6.**
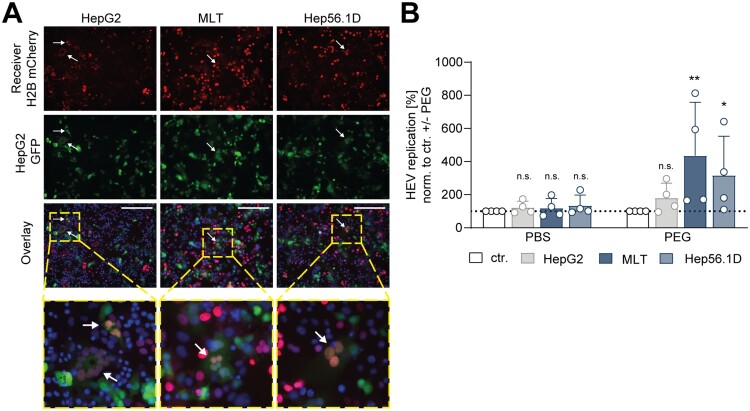
Interspecies heterokaryon formation promotes HEV-3 replication. (A) Fusion of GFP-expressing HepG2 cells (green) transfected with a HEV-3 subgenomic reporter replicon to either H2B-mCherry (red) expressing naïve HepG2 or murine hepatic cells (MLT or Hep56.1D) using polyethylene glycol (PEG). Representative immunofluorescence images of n = 4 at 24 h post fusion. Scale bar = 200 µm. (B) HEV replication was quantified by luciferase readout at 24 h post fusion. HEV-transfected HepG2 cells without co-cultivation were used as a control (ctr.) and for normalization (norm.). Mean and individual values + SD from n = 4 experiments are shown. Statistical significance was calculated on log transformed data using an ordinary two-way ANOVA with Dunnett multiple comparison correction. N.s., not significant with *p*-value >0.05; * = *p*-value <0.05; ** = *p*-value <0.01.

Since antiviral host proteins are not necessarily regulated by IFNs, we next examined if the intrinsic expression of restriction factors contributes to the murine species barrier to HEV infection. Therefore, we generated interspecies heterokaryons via polyethylene glycol (PEG)-mediated cell fusion, as previously done in HCV research [35,45]. For this, HepG2 cells, labelled by cytoplasmic expression of green fluorescent protein (GFP), were transfected with HEV-3 subgenomic replicons. Afterwards, HepG2 cells with active HEV-3 replication were fused with human or murine hepatic cells, expressing H2B-linked mCherry in the nucleus. Upon successful fusion of both cell populations, heterokaryons with mCherry expression in the nucleus will additionally express GFP in the cytoplasm (Figure 6(A)). Quantification of the secreted Gaussia luciferase demonstrated comparable HEV-3 replication in the phosphate-buffered saline (PBS)-treated control co-cultures and enhanced HEV-3 replication in co-cultures treated with PEG (Figure 6(B)). Similarly, enhanced HEV replication fitness was detected when HEV-replicating murine cells were fused to naïve HepG2 cells (Figure S8). Collectively, these observations suggest that the murine species barrier to HEV replication is defined by the absence of host dependency factors or incompatibility of murine orthologues, rather than by the presence of constitutively expressed or virus-induced dominant HEV restriction factors interfering with viral replication.

### Post attachment steps of HEV-3 entry differ between murine and human hepatocytes

**Figure 7.**
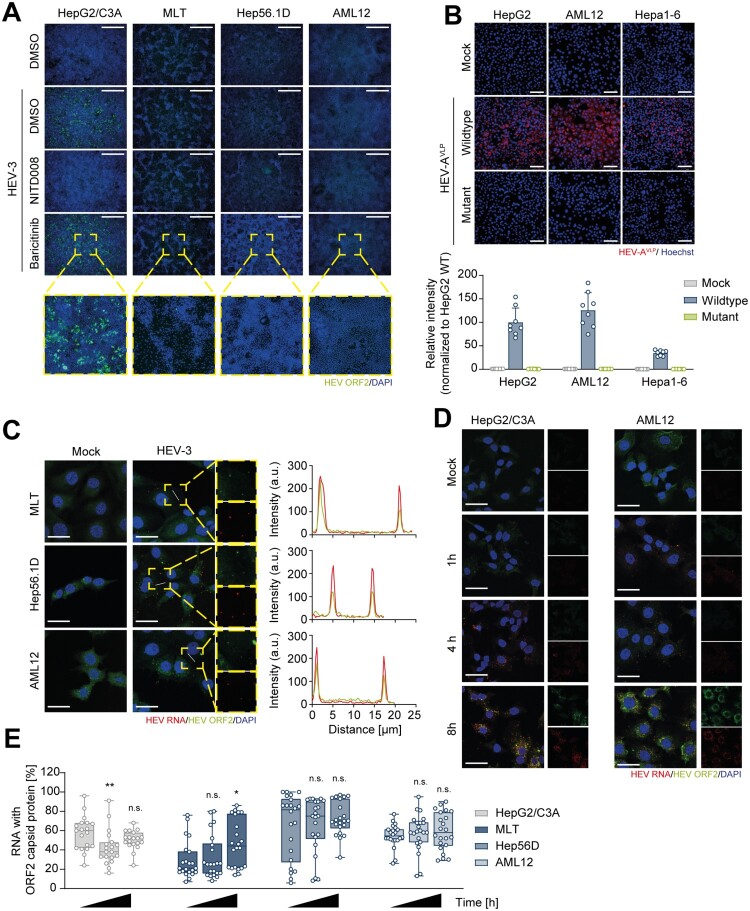
Entry of infectious HEV-3 into murine liver cells is blocked after viral attachment. (A) Representative immunofluorescence images. HEV capsid protein was stained in human HepG2 and murine MLT, Hep56.1D and AML12 cells at 5 days post HEV-3 inoculation (n = 3). Cells were either (pre-)treated with baricitinib [2 µM], NITD008 [0.5 µM] or with DMSO as solvent control. ORF2 capsid protein = green; DAPI = blue; scale bar = 1000 µm. (B) Binding of HEV virus-like particles (VLPs) of the species *Paslahepevirus balayani* (HEV-A) to the human liver cell line HepG2 and murine hepatic cell lines (AML12 and Hepa1-6) was measured by immunofluorescence assay. The relative fluorescent intensity was quantified by ImageJ and normalized to HepG2 cells incubated with wildtype (WT) VLPs. Representative images and mean values as well as individual values + SD from n = 2 experiments with 4 technical replicates are shown. VLPs = red; nuclei = blue; scale bar = 50 µm. (C) Binding of infectious HEV-3 particles to murine hepatic cell lines after 2 h incubation at 4 °C. HEV capsid protein was detected by immunofluorescence staining and HEV RNA by RNAscope. Representative images of n = 3 independent experiments are shown. Line graphs show the fluorescence intensities of HEV RNA and ORF2 capsid protein staining measured across the region of interest indicated by the white line in the immunofluorescence images. HEV RNA = red; HEV ORF2 capsid = green, DAPI = blue; scale bar = 50 µm. (D) Visualization of viral particle internalization in human HepG2/C3A and murine AML12 cells via HEV ORF2 capsid protein immunofluorescence staining and RNAscope for detection of HEV RNA over the indicated time frame. Representative images of n = 3 independent experiments are shown. HEV RNA = red; HEV ORF2 capsid protein = green, DAPI = blue; scale bar = 50 µm. (E) Quantification of detected HEV RNA associated with ORF2 capsid protein per nucleus from panel C and supplementary figure 6. Five to eight frames per biological replicate were analysed per condition. Individual values per analysed frame are shown. Statistical significance was calculated on single values per cell line using ordinary one-way ANOVA with Dunnett multiple comparison correction. N.s., not significant with *p*-value >0.05; * = *p*-value <0.05; ** = *p*-value <0.01.

Based on our findings, we hypothesized that the inability of HEV to interact with murine orthologues of viral entry proteins or the absence or low expression of such receptors, represents a significant determinant limiting HEV infection in murine hepatocytes. Consequently, we first investigated whether the inoculation of murine hepatic cells with infectious, cell culture-derived HEV-3 results in productive infection. In line with the PMH data (Figure 1(B)), no specific ORF2 capsid protein staining was detectable upon inoculation of murine hepatic cells with infectious HEV-3 (Figure 7(A)). Similarly, pre-treatment with baricitinib did not affect susceptibility of murine cell lines. To dissect the molecular mechanisms underlying the hypothesized failure of HEV-3 entry into murine cells, we further examined the individual steps of viral entry. First, to exclude insufficient expression of previously characterized HEV cofactors implicated in viral entry as a potential limitation, we conducted RNA-seq analysis, which revealed that currently known entry factors were expressed at adequate levels in murine cells (Figure S9). Subsequently, we assessed viral attachment to murine cells using a virus-like particle (VLP) system, which mimics authentic virus binding and penetration into host cells [46]. As previously demonstrated, VLPs derived from the species *Paslahepevirus balayani* (HEV-A), show strong binding affinity to HepG2 cells, whereas VLPs with an alanine substitution at position N562A and T564A at the surface of the P domain of the ORF2 capsid protein failed to attach (Figure 7(B)). Notably, the wildtype VLPs also showed high affinity for the murine AML12 cell line and, to a lesser extent, for an additional murine cell line Hepa1-6. Combining the recently established RNA *in situ* hybridization assay [47] with an immunofluorescence staining of the HEV ORF2 capsid protein, we could confirm viral attachment to murine cells upon inoculation with infectious viral particles, as shown by detection of HEV RNA associated with capsid protein after intensive washing post inoculation (Figure 7(C)). Prolonged incubation at 37°C resulted in increasing signal for both viral RNA and capsid protein in all tested human and murine cell lines (Figure 7(D) and S10). However, while detectable RNA-capsid protein association gradually decreased over time in HepG2/C3A cells, murine cells showed sustained association of viral RNA with capsid protein, providing evidence for a block in viral particle entry mechanisms, such as viral particle uptake post-attachment or uncoating, in murine cells (Figure 7(E)). To pinpoint the exact step of viral entry impediment, we examined whether murine cathepsin L (CTSL)-mediated proteolytic processing of the HEV ORF2 capsid protein, a process suggested to facilitate viral RNA release into the cytosol in human cells [48], is functional. An *in vitro* CTSL cleavage assay showed the presence of a cleavage product, which appearance can be inhibited by treatment with the cysteine protease inhibitor K11777 (Figure S11). Based on these observations, we hypothesized that the restriction might instead occur at an earlier stage, such as viral trafficking to the endolysosomal compartment. However, RNA-FISH combined with staining for human or murine lysosomal markers showed that murine cells not only contained a higher number of lysosomes but also exhibited a significantly greater proportion of viral RNA signal overlapping with lysosomal markers (Figure S12). These findings suggest that delivery of viral RNA to the endolysosomal pathway is not impaired in murine cells, making this step unlikely to represent the restrictive barrier. In summary, our results indicate that while HEV-3 particles attach to murine hepatocytes and enter the endolysosomal pathway, these cells remain refractory to HEV-3 infection due to a block in the entry process.

## Discussion

Among the five known human hepatitis viruses (HAV, HBV, HCV, HDV and HEV), HEV of the species *Paslahepevirus balayani* displays the broadest species tropism. HEV-3 has been detected in pigs, wild boar, rabbits, and deer, while natural infection of the other human hepatitis viruses is restricted to primates. Interestingly, infections with the distantly related rat HEV have recently been observed in humans, raising significant global health concerns [49,50]. Jo et al. recently proposed a rodent origin for human-pathogenic HEV [51]. In line with this hypothesis, several studies demonstrated that Mongolian gerbils are susceptible to HEV-1, −3, and −4 as well as rat HEV [52–55], suggesting low species barriers to HEV infection among rodents. However, the Rodentia order is remarkably diverse, containing over 2,000 separate species [56], and permissiveness of mice for HEV infection remains debated and hampers the development of an immunocompetent, genetically modifiable small animal model.

Here, we report that three different murine hepatic cell lines support HEV-3 replication, protein expression and subsequently the assembly and release of infectious HEV-3 particles upon transfection of viral RNA (Figures 2 and 3). These results indicate that murine orthologues of key host dependency factors for RNA replication and infectious particle production can be successfully exploited by HEV. Although these steps of the viral replication cycle can be completed *in vitro*, the low levels of sustained replication, especially beyond 3 days post transfection (Figure S2), indicate that murine hepatocytes do not efficiently support HEV replication. This incomplete cellular competence may contribute to the limited cross-species transmission of HEV and may act as a secondary restriction beyond other established species barriers.

Determinants of viral host tropism do not rely solely on exploitation of host factors, but also on viral evasion from cellular antiviral mechanisms including antiviral effector proteins. These restriction factors are often induced by IFN signalling as mediators of the cell-intrinsic immune response. However, through evolutionary pressure, viruses evolved viral antagonists that limit these antiviral defenses in their natural host and contribute to viral persistence. For HEV, viral proteins encoded by all three ORFs have been suggested to antagonize type I IFN-mediated responses, leading to a weak induction of ISG expression and partial IFN resistance in human cells [43]. As previously shown by Todt et al. [57], we were able to confirm that HEV-3 replication is able to restrict the induction of ISGs in human cells (Figures 4(B,C) and S4). However, upon spillover events to a non-natural host, viruses frequently encounter innate immune responses they are not evolutionarily adapted to. The innate immune system can vary substantially between host species and consequently viral antagonists may fail to suppress immune responses in the new host, preventing establishment of a viral infection. In line with this, we observed in murine cells an increased sensitivity of HEV-3 to IFN-α treatment, which correlates with elevated antiviral gene expression and the absence of HEV-mediated downregulation of ISG induction (Figure 4). Increased sensitivity might result from the impaired ability of HEV-3 to dampen IFN signalling, potentially due to insufficient expression levels of viral antagonists resulting from lower replication efficiency in the murine host (Figure 2(B) and Figure 3(A)). Alternatively, the diminished antagonism might be due to an evolutionary lack of adaptation of HEV to the murine innate immune system emphasizing that mouse hepatocytes do not fall within the natural host tropism of HEV. Even in porcine cells, which represent a reservoir species for HEV-3 infection, recent work by Schlienkamp, Veiga et al. [19] demonstrated that a functional IFN response is a key determinant in restriction of HEV-3 infection in species other than humans and likely reflects differences in disease outcomes seen in swine and humans [19,58]. Unexpectedly, treatment with the JAK inhibitor baricitinib had only a minor impact on permissiveness of murine cells (Figure 5(A)). The following investigations further imply that HEV-3 replication seems to be not effectively sensed by the innate immune system of murine cell lines, as evidenced by low to undetectable ISRE-dependent promoter activity, minimal induction of ISG expression following transfection of HEV-3 RNA and no impact of MAVS knockout on viral replication (Figures 5(B–D), S6 and S7). We acknowledge the fact that viral replication levels are significantly lower in murine cells, possibly influencing the strength of innate immune response induction. However, we are confident that the electroporation-mediated transfection of viral RNA delivers sufficient quantities of pathogen-associated molecular patterns (PAMPs) to murine cells. Although the expression of the majority of restriction factors is IFN-regulated, a small portion exhibit high basal expression levels, contributing to intrinsic resistance to viral infections independent of IFN induction. One such candidate might be CD302: While it remains unknown whether murine CD302 also exerts antiviral activity against HEV infection in murine hepatocytes, its human ortholog has been identified as an HEV restriction factor [59]. To further investigate the functional relevance of such a restriction factor in limiting HEV replication in murine cells, we generated interspecies heterokaryons between human and murine cells. As HEV-3 replication in human cells was not restricted following fusion with murine cells (Figure 6(B)), we hypothesize that the limited replication of HEV-3 in murine cells is likely due to the absence, low expression, or incompatibility of non-essential host dependency factors, rather than to the presence of a dominant restriction factor.

Lastly, the host range of viruses has been considered to be predominantly determined at the level of virus entry, with the compatibility of host cellular entry factors and viral receptor-binding proteins being highly important. However, recent literature suggests that this compatibility is often given, with viral genes involved in entry being rarely subjected to selection upon zoonotic transmission [60,61]. Certain viruses, such as human-restricted HBV, HDV and HCV, appear to deviate from this observation, as viral entry plays a significant role in their host range determination [37,62,63]. Although the *bona fide* entry receptor for HEV has yet to be identified, viral entry is likely also a key factor in restricting HEV host tropism. This is underscored by the observation that VLP from only the human-infecting HEV species of the genera *Paslahepevirus* and *Rocahepevirus* showed binding affinity to and particle uptake from human cells [46]. Interestingly, although murine cell lines as well as PMH were refractory to HEV-3 infection (Figures 1 and 7), we present evidence that attachment of HEV-3 particles to murine hepatocytes is feasible (Figure 7(B, C)). With prolonged incubation times, we observed decreasing amounts of RNA associated with HEV capsid protein in HepG2/C3A cells (Figure 7(D,E)), most likely due to internalization and uncoating of viral particles in human hepatocytes [47]. Interestingly, for murine hepatic cells we observed a strong increase, especially in the viral RNA signal at 8 h post inoculation, with sustained association of viral RNA with the viral capsid protein. Proteolytic cleavage of the ORF2 capsid protein by cathepsins during trafficking through the endolysosomal pathway is suggested to aid capsid disassembly and likely also facilitate downstream activities required for complete viral uncoating of naked HEV particles in human cells [47,48]. Although we observed cleavage upon incubation of recombinant ORF2 capsid protein with recombinant murine CTSL (Figure S11), incomplete or premature pH- as well as proteolytic-dependent capsid modifications may still impede correct signalling cascades during the viral uncoating step in murine hepatocytes. Our findings suggest that the entry of naked HEV particles is impeded in murine hepatocytes, resulting in murine liver cell lines being refractory to HEV-3 infection. In line with this, we detected only low levels of intracellular viral RNA at 72 h post inoculation and a complete absence of transcriptomic signatures in PMH following inoculation with infectious HEV-3 (Figure 1(D, E)) suggesting unsuccessful uptake of infectious virions as well. Consequently, we propose that murine susceptibility to HEV infection is restricted by a post-attachment, pre-replication blockage of the viral replication cycle.

In summary, we provide the first mechanistic evidence that murine hepatocytes support HEV-3 replication and release of infectious particles, albeit with limited efficiency due to restrictions that are independent of the innate immune response. Most notably, our findings demonstrate that murine hepatocytes are refractory to HEV-3 infection, as the viral replication cycle is fundamentally impaired by a block at the stage post attachment and before viral replication is initiated. These insights significantly advance the understanding of host specificity and cross-species barriers in zoonotic HEV infection.

## Supplementary Material

GraphicalAbstract1.tif

Supplementary_Data_revised_clean.pdf
