# Supplementary figures and images for: Viral entry defines the hepatitis E virus species barrier in murine hepatocytes
Source: Emerg Microbes Infect. 2026 Jul 27;15(1):2706321. doi: 10.1080/22221751.2026.2706321 (PMC13410553; doi:10.1080/22221751.2026.2706321)

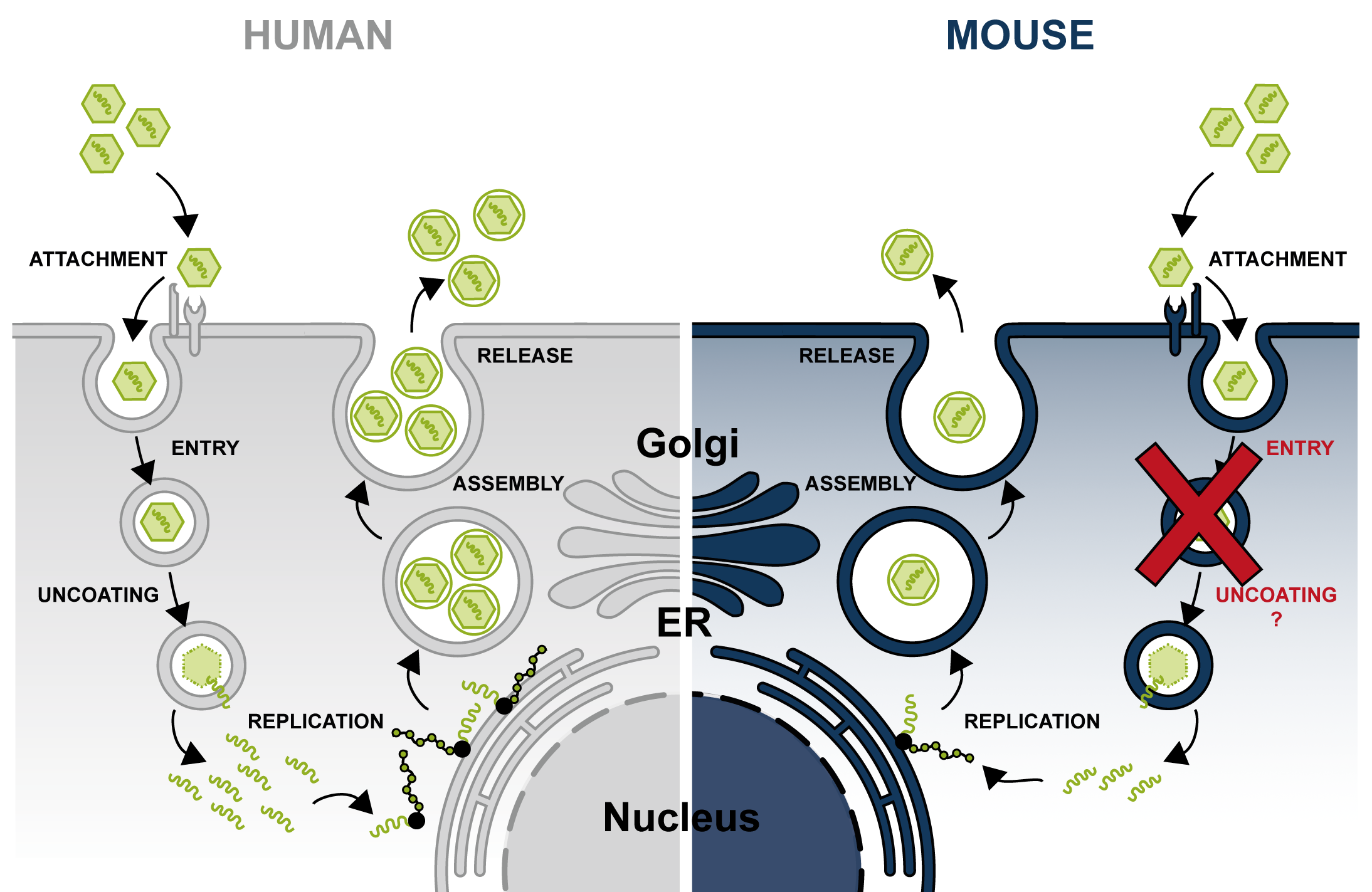

Supplement: GraphicalAbstract1.tif [file TEMI_A_2706321_SM0166.tif]
