# Supplementary material for: Viral entry defines the hepatitis E virus species barrier in murine hepatocytes
Source: Emerg Microbes Infect. 2026 Jul 27;15(1):2706321. doi: 10.1080/22221751.2026.2706321 (PMC13410553; doi:10.1080/22221751.2026.2706321)
Supplement: Supplementary_Data_revised_clean.pdf [file TEMI_A_2706321_SM0165.pdf]

Supplementary Information

Viral entry defines the hepatitis E virus species barrier in murine hepatocytes

Nicola Frericks[1,2], Olinda Pinto Veiga[3], Leyla Sirkinti[2,4], Hoang Duy Nguyen[5], Tina Sliwinski[1,2], Hongbo Guo[6], Yannick Brüggemann[1,2], Thomas Burkard[1], Rainer G. Ulrich[7], Richard J.P. Brown[2,4], Tran Tuoc[5], Huu Phuc Nguyen[5], Wenshi Wang[6], Daniel Todt[2,4,8], Volker Kinast[3], Eike Steinmann[1,2*]

[1] Department of Molecular and Medical Virology, Ruhr University Bochum, Bochum, Germany

[2] Hepatitis E Virus Research Hub (HepE-Hub), Bochum, Germany

[3] Institute for Medical Microbiology and Virology, Carl von Ossietzky University Oldenburg, Oldenburg, Germany

[4] Department of Translational and Computational Infection Research (TRACiR), Ruhr University Bochum, Bochum, Germany

[5] Department of Human Genetics, Ruhr University Bochum, Bochum, Germany

[6] Department of Pathogen Biology and Immunology, Jiangsu Key Laboratory of Immunity and Metabolism, Jiangsu International Laboratory of Immunity and Metabolism, Xuzhou Medical University, Xuzhou, China

[7] Institute of Novel and Emerging Infectious Diseases, Friedrich-Loeffler-Institut, Greifswald-Insel Riems, Germany

[8] European Virus Bioinformatics Center (EVBC), Jena, Germany

Supplementary material and methods

Cell culture

HepG2 (ATCC-NR.: HB-8065), Hep56.1D, MLT and MLT 5H MAVS -/- [1] (all three kindly provided by Thomas Pietschmann, Twincore, Hannover, Germany), HEK293T (ATCC-Nr.: CRL-3216) and Hepa1-6 (ATCC-NR.: CRL-1830) were cultured in Dulbecco's Modified Eagle's Medium (DMEM, Gibco, Thermo Fisher Scientific) supplemented with 10% (v/v) fetal calf serum (FCS, GE Healthcare), 1% (v/v) MEM non-essential amino acids (NEAAs, Gibco, Thermo Fisher Scientific), 100 U/mL penicillin and 100 µg/mL streptomycin (Gibco, Thermo Fisher Scientific), and 2 mM L-glutamine (Gibco, Thermo Fisher Scientific). MLT cells were cultivated in medium further supplemented with 5 µg/mL puromycin (Sigma-Aldrich). For MLT 5H MAVS-/- further 5 µg/mL blasticidin (BioReagents, Fischer Scientific), and 750 µg/mL G418 (Biochrom) were added to the cell culture media. AML12 cells (ATCC-Nr.: CRL-2254) were maintained in DMEM/Ham's F-12 medium (Gibco, Thermo Fisher Scientific) supplemented with 10% FCS, 40 ng/mL dexamethasone (Merck), 10 µg/mL insulin, 5.5 µg/mL transferrin, 6.7 ng/mL selenium (ITS-X, Gibco, Thermo Fisher Scientific), 100 U/mL penicillin and 100 µg/mL streptomycin. HepG2/C3A cells (kindly provided by Charles Rice, The Rockefeller University, New York, USA) were cultured in Eagle's minimum essential Medium (MEM, Gibco, Thermo Fisher Scientific) supplemented with 10% (vol/vol) ultralow IgG FCS (Gibco, Thermo Fisher Scientific), 100 µg/mL gentamicin (Gibco, Thermo Fisher Scientific), 2 mM L-glutamine (Gibco, Thermo Fisher Scientific), 1 mM sodium pyruvate (Gibco, Thermo Fisher Scientific), 1% (v/v) NEAAs (Gibco, Thermo Fisher Scientific). HepG2-derived cell lines were cultured on rat collagen-coated (SERVA Electrophoresis) and HEK293T on poly-L-lysine-coated (Sigma-Aldrich) cell culture vessels.

Primary mouse hepatocytes (PMH) (Lot MH211103 Strain CD-1, Lot MH210923 strain CD-1, Lot MH190923 strain CD-1, Lot MH240111-2 strain Black 6 (C57BL6JR)) and PHH (Lot HH250124) were purchased from Primacyt (Schwerin, Germany) as cryopreserved hepatocytes and thawed and seeded on 24-well plates according to the manufacturer's instructions.

Plasmids

The plasmids encoding Kernow-C1/p6 (HEV-3; GenBank accession Nr.: JQ679013), its derivative as well as a Sar55/S17 (HEV-1; GenBank accession no. AF444002, [2]) plasmid encoding a Gaussia

luciferase reporter were kindly provided by Suzanne U. Emerson (National Institute of Allergy and Infectious Diseases, United States of America). The plasmids containing the strain HEV 83-2-27 sequence (HEV-3; GenBank accession Nr.: AB740232) and its Gaussia luciferase reporter derivative were kindly provided by Takaji Wakita (National Institute of Infectious Diseases, Japan). pCMVR8.74 (Addgene plasmid #22036) and pMD2.G (Addgene plasmid #12259) were a gift from Didier Trono (École Polytechnique Fédérale de Lausanne, Switzerland). The JFH1 sub genomic replicon harboring a firefly luciferase was previously described [3]. The pLVX-M-puro plasmid (Addgene plasmid #125839) was a gift from Boyi Gan (The University of Texas MD Anderson Cancer Center, United States of America). The pLVX-M-puro vector was modified by inserting an enhanced GFP (eGFP) under the control of the human cytomegalovirus (CMV) promoter by using the Gibson Assembly approach. The plasmid pLenti6-H2B-mCherry (Addgene plasmid #89766) was a gift from Torsten Wittmann (University of California, United States of America). The ISRE-reporter plasmid was cloned by inserting the sfGFP fused to a mouse ornithine decarboxylase (MODC) protein degradation domain under the control of seven elements of ISRE [4] and a minimal CMV promoter into the pLentiPGK Puro DEST JNK KTR Clover plasmid (Addgene plasmid #59151), which was a gift from Markus Covert (Stanford University, United States of America). All cloning strategies and plasmid sequences are available upon request.

Generation of stable cell lines

Stable cell lines were generated by lentiviral transduction. For lentiviral pseudoparticle production, HEK293T cells were seeded at a density of 4×10^5 in 6-well format or $1.5\text{x}10^6$ cells on PD60. The day after, OptiMEM (Gibco, Thermo Fisher Scientific), Lipofectamine 2000 (Invitrogen), plasmids encoding the gene of interest with the lentiviral packaging and envelope-expressing plasmids pCMVR8.74 and pMD2.G (ratio 1:1:1) were mixed and applied on HEK293T cells as recommended by the manufacturer's instructions. At 16 hours post transfection, medium was changed and lentiviral particles harvested at 48 hours and 72 hours post transfection. Lentiviral particles were combined from both harvesting time points, filtered (0.45 µm) and supplemented with 50 mM Hepes (Gibco, Thermo Fisher Scientific) and 4 µg/mL polybrene (Sigma-Aldrich) prior to storage at -80 °C. For transduction, HepG2 cells (5×10^5) and murine cell lines (3×10^5) were seeded on a 6-well plate. The following day,

cells were inoculated with 0.5 mL of lentiviral particles for 16 h. Selection of transduced cells was started 48 hours post transduction using 2.5 µg/mL (HepG2) and 5 µg/mL (MLT, Hep56.1D) puromycin or 5 µg/mL blasticidine, depending on the antibiotic resistance encoded by the plasmid. Expression was validated via fluorescence microscopy.

Production of cell culture-derived HEV (HEVcc)

Production of infectious, cell-culture derived HEV was done with some minor protocol adjustments to the ones previously described [5,6]. Shortly, 5 µg of full-length RNA of Kernow-C1/p6 plasmid DNA was generated by *in vitro* transcription and transfected into 5x10^6 human or murine cells by electroporation. Viral protein production after electroporation was confirmed by seeding cells (1x10^5 cells/well) in 24-well plate format and an immunofluorescence staining for ORF2-encoded capsid protein at indicated time points. For harvesting enveloped HEVcc, cell culture supernatant was collected from PD100, filtered (0.45 µm) at indicated time points and stored at 4 °C until quantification of the virus titer was done. Non-enveloped (naked) HEVcc was harvested from the cell lysate, by washing the cells once with PBS followed by scraping them from the surface of the cell culture vessel and diluting them in the respective cell culture medium. Cells were pelleted at 200 x g for 5 min and resuspended in 1 mL per electroporation of the respective cell culture medium prior to three freeze/thaw cycles using liquid nitrogen and ice. Cell debris were removed by a centrifugation step at 10,000 x g for 10 min at 4 °C. The supernatant was aliquoted and stored at -80 °C. Viral titers were calculated after performing a serial dilution assay using HepG2/C3A cells and counting the focus forming units (FFU)/ml.

Gaussia luciferase reporter assay

To assess HEV replication fitness in murine cells compared to human cells, 5x10^6 cells were transfected with 5 µg of *in vitro* transcribed HEV sub genomic replicon RNA, carrying a Gaussia luciferase, as described for HEVcc production. After electroporation 2x10^4 cells/well were seeded in a 96-well plate. At 4 hours post transfection, fresh medium with and without Ribavirin (50 µM final concentration) was added as a control. In order to quantify the secreted luciferase in relation to HEV replication, cell culture supernatant was harvested at 4, 24, 48,72, 96 and 120 hours post transfection and stored at 4 °C until measurement. For Gaussia luciferase activity measurement, 20 µl of the collected supernatant was transferred to a white, flat-bottom 96-well microplate (Greiner Bio-One) prior to automatic addition of

0.2 µmol/L coelenterazine in PBS (Carl Roth) and measurement using the Centro XS3 LB 960 luminometer (Berthold Technologies).

Firefly luciferase reporter assay

For the quantification of the HCV replication fitness in murine cells in presence or absence of RIG-I-like receptor dependent signaling, $5x10^6$ MLT and MLT 5H MAVS -/- cells were transfected with 5 µg of *in vitro* transcribed HCV JFH1 sub genomic replicon RNA, carrying a firefly luciferase gene, as described earlier. After electroporation, $2x10^4$ cells/well were seeded in a 96-well plate. At 4 hours post transfection, fresh medium with and without Ribavirin (50 µM final concentration) was added as a control. In order to quantify the luciferase activity in correlation to HCV replication, at 4, 24, 48 and 72 hours post transfection the cell culture supernatant was removed and cells lysed on the plate by addition of 35 µL/well luciferase lysis buffer (1% Triton X-100, 25 mM diglycine, 15 mM MgSO4, 4 mM EGTA, and 1 mM DTT). Samples were stored at −20°C until luciferase measurement was performed. After sample thawing, 20 µl cell lysate per well were transferred to a white 96-well luminometer plate preloaded with 72 µL of assay buffer (25 mM diglycine, pH 7.8; 15 mM potassium phosphate; 15 mM $MgSO_4$; 4 mM EGTA, pH 8; 1 mM DTT; and 2 mM ATP, pH 7.8). Subsequently, 40 µL of luciferin solution (0.14 mM D-luciferin, 25 mM diglycine) was automatically added, and bioluminescence was measured using the Centro XS3 LB 960 luminometer (Berthold Technologies).

HEV infection assays

For infection assays, HepG2/C3A ($5x10^4$/well) and MLT, Hep56.1D and AML12 ($1x10^4$/well) cells were seeded into 24-well plate format. The seeding of PMH and primary human hepatocytes (PHH) was performed according to manufacturer's instruction. At the time of cell seeding, pre-treatment with 2 µM Baricitinib (Selleckchem, Medchem Express) or vehicle control (dimethyl sulfoxide, DMSO) was added. At least 16 hours after pre-treatment, cells were inoculated with HEVcc at a multiplicity of infection (MOI) of 1 for hepatoma cell lines. For PMH and PHH, the inoculum corresponded to 10% of the culture volume using a virus stock with a titer of $2.5\text{-}3x10^5$ FFU/ml, resulting in an estimated MOI of 1.5–2, depending on the recommended cell seeding density. The following day, cell culture supernatant was removed and cells washed once with PBS prior to addition of fresh cell culture medium containing 0.5 µM NITD008 (MedchemExpress), 2 µM baricitinib (Selleckchem, Medchem Express)

or DMSO (0.2% final concentration) as a vehicle control. Cells were fixed using 3% paraformaldehyde (PFA) and subjected to immunofluorescence staining after 5 days of incubation. For primary hepatocytes, potentially occurring intracellular virus was harvested by three freeze-thaw cycles in 100 µl PBS as described above. Viral infectivity was determined by titration on HepG2/C3A cells and FFU/mL calculation.

IFN treatment of PMH

To validate the immunoreactivity of PMH, PMH seeded for HEV infection assays were treated with 100 IU/ml of murine IFN-α (Miltenyi Biotec) in parallel. Treated and untreated PMH were harvested at 6, 12 and 48 h post treatment. For this, the cell culture supernatant, was removed and cells washed once with PBS prior to cell lysis as described in the RNA sequencing section.

Dose response assay

To evaluate the HEV sensitivity to IFN-α treatment in murine cells versus human cells, cells were transfected with *in vitro* transcribed HEV subgenomic replicon RNA and seeded with 50 µl/well into 96-well plates as described above. At 4 hours post transfection, 50 µl cell culture medium that contained serial dilutions of the species-specific IFN-α (human: IntronA, subtype interferon alfa-2b; murine: Miltenyi Biotec) at 2x final concentration (starting at 3,000 IU/mL final concentration for human and 100 IU/mL final concentration for murine) was added. Each concentration was tested in six technical replicates. After incubation for 3 days, cell culture supernatant was harvested for Gaussia luciferase activity measurement and cells subjected to a cell viability assay.

Evaluation of the immunoreactivity of murine and human liver cell lines

To characterize the immunoreactivity of the used murine and human liver cell lines, $1x10^5$ cells/well were seeded into 24-well format. The following day, cells were either untreated, transfected with 0.5 µg poly(I:C) using lipofectamine 3000 according to the manufactures instructions or treated with 1,000 IU/ml of species-specific IFN-α. After 16 h of incubation, cells were lysed for RNA extraction using the NucleoSpin RNA kit (Macherey-Nagel), followed by cDNA synthesis (PrimeScript RT Master Mix, Takara) and real-time quantitative PCR to measure ISG expression.

Modulation of the IFN pathway by IFN and Janus kinase inhibitor treatment

To investigate the impact of IFN signaling on HEV replication and virus production, cells electroporated with either *in vitro* transcribed full-length or subgenomic Kernow-C1/p6 RNA were treated < 4 hours post transfection with 2 µM ruxolitinib (Selleckchem), 2 µM baricitinib (Selleckchem, Medchem Express), 100 IU/mL IFN-α or DMSO (final concentration. 0.02%) as a vehicle control. Replication under IFN pathway modulation was quantified using the Gaussia luciferase reporter assay. Impacts on viral particle production were assessed by virus harvesting and performance of a serial dilution assay as described above. The drug activity was validated by cDNA synthesis (PrimeScript RT Master Mix, Takara) followed by real-time quantitative PCR to measure ISG expression. Cytotoxicity of drug treatment was monitored by a cell viability assay.

Innate immune response to HEV infection under IFN treatment

To study the cellular response upon IFN treatment during HEV infection, $2.5x10^6$ cells were transfected with 2.5 µg full-length Kernow-C1/p6 RNA or tRNA as described above. Electroporated cells were seeded at a density of $2x10^5$ cells/well of a 24-well plate. Two days post transfection, cells were left untreated or treated with 100 IU/mL of IFN-α (human: IntronA, subtype interferon alfa-2b; murine: Miltenyi Biotec). After 6 hours of incubation, cells were lysed and subjected to RNA extraction for cDNA synthesis (PrimeScript RT Master Mix, Takara) and real-time quantitative PCR-based and RNA-seq-based quantification of ISG expression.

ISRE-GFP reporter assay

To monitor ISRE activity upon HEV replication, ISRE-GFP reporter cell lines were electroporated with *in vitro* transcribed full-length Kernow-C1/p6 RNA or tRNA as previously described. Electroporated as well as naïve cells were seeded into 24-well ($1x10^5$ cells/well) or 6-well ($1x10^5$ cells/well) plates. Three days post electroporation, cells in 24-well format were fixed and subjected to immunofluorescence staining, whereas cells cultivated in 6-well format were analyzed by flow cytometry. Cells treated with 100 IU/mL of IFN-α (human: IntronA, subtype interferon alfa-2b; murine: Miltenyi Biotec) for 16 hours prior to fixation with 3% PFA and flow cytometry were used as a control for ISRE-dependent GFP expression.

Heterokaryon fusion assay

Prior to induction of cell fusion, HepG2 GFP cells were electroporated as described earlier for the HEV replication assay. Transfected cells were transferred to 6 mL of cell culture medium (cell density 8x10^5 cells/ml). Cell suspensions of human and murine cells expressing H2B mCherry were diluted to 2x10^5 cells/ml. After combination of 1.5 mL of HEV replicon transfected HepG2 GFP cells with 1 mL of mCherry expressing cells, 1 ml/well of the cell suspension was seeded into 24-well format. As a control, human and murine cells expressing H2B mCherry were electroporated with HEV RNA using the same protocol. The electroporated cells were then diluted to 2x10^5 cells/ml of which 1.5 ml was mixed with 1 ml of naïve HepG2 GFP cells (8x10^5 cells/ml). The resulting co-culture was seeded into each well of a 24-well plate with 1 ml/well. After 24 hours of incubation at 37 °C and 5% CO2, a cell density should be about 80% for induction of cell fusion. The cell culture supernatant was aspirated and co-cultured cells washed once with 1 mL PBS prior to addition of 500 µl pre-warmed 40% PEG-1500 (Roche) or PBS as a control. After incubation for 5 min at room temperature, the PEG or PBS was carefully removed, and cells washed five times with 1 mL PBS for 1 min per wash step. After washing, 1 mL of pre-warmed cell culture medium was added to the cells. After 24 hours of incubation at 37 °C and 5% CO_2 supernatant was collected and subjected for the quantification of HEV replication via the Gluc replication assay. At the same time, cells were fixed in 3% PFA and heterokaryon formation assessed by fluorescence microscopy.

Cell binding assay

To investigate the ability of VLPs to attach to mouse cells, VLPs of the species *Paslahepevirus balayani* (HEV-A) were generated and a cell binding assay was performed as previously described [7]. The HEV-A VLPs with N562A and T564A mutations (M3) served as an control. In short, cells were washed with pre-chilled PBS, when confluency reached 80%, and then incubated with wild type or mutant VLPs (40 µg/mL) or PBS (negative control, NC) for 1 hour at 4 °C with slow shaking on a rotator. After removing the supernatant, cells were extensively washed with ice-cold PBS for 3-4 times to remove unbound VLPs. For immunoflourescence staining, cells were fixed with 4% PFA and then incubated with antibodies diluted in 2% bovine serum albumin (BSA). His-tag-specific mouse monoclonal antibody (66005-1-Ig, Proteintech) and CoraLite594-conjugated Goat anti-mouse IgG(H+L) (SA00013-3,

Proteintech) were used as first and secondary antibody, respectively. Nuclei were stained with Hoechst Stains (Invitrogen, USA). Fluorescent images were captured by Olympus fluorescent microscope (model U-LH100HGAPO). For each independent experiment, the fluorescent images were taken from random areas with the same parameters set on the microscopy (e.g., image resolution, imaging speed and acquisition time). For each image, the fluorescence intensity of two independent channels (one for the nucleus, the other for the detected protein) were quantified separately by ImageJ software. The fluorescent intensity of the detected protein was further normalized by its nucleus fluorescent intensity to exclude the possible interference of varied cell numbers captured in the images.

Cell viability assay

For the assessment of cell viability after drug treatment a MTT (3-(4,5-dimethylthiazol-2-yl)-2,5-diphenyltetrazolium bromide) assay or CytoTox 96® Non-Radioactive Cytotoxicity Assay (Promega) was performed. For the MTT assay, prewarmed 0.5 mg/mL of MTT substrate (Sigma -Aldrich) mixed in cell culture media was applied to the cells and incubated at 37 °C with 5% CO2 for 1-2 h. The reaction was stopped by removal of the cell culture supernatant and addition of 50 µl DMSO per well of a 96-well plate. Absorbance was measured at 570 nm using a Sunrise absorbance microplate reader (Tecan Group Home). Cells treated with 70% ethanol for 10 min were used as background control. Each concentration was tested in at least three technical replicates For MLT cells, cell viability was measured using the CytoTox 96® Non-Radioactive Cytotoxicity Assay (Promega) according to manufacturer's instruction. Shortly, the release of the lactate dehydrogenase into the supernatant was measured by incubation of 50 µl/well of supernatant with equal amounts of CytoTox 96 reagent in 96-well format. After incubation for 30 min protected from light, the reaction was stopped by addition of 50 µl of the stop solution and direct absorbance measurement at 492 nm on the Sunrise microplate reader (Tecan).

Immunofluorescence staining

For immunofluorescence staining, the supernatant was aspirated and cells fixed in 3% PFA for at least 20 min. Cells were washed three times with PBS prior to permeabilization using 0.2% Triton X-100 for 5 min. After three additional PBS washes, cells were incubated in 5% horse serum in PBS for 1h at room temperature. HEV capsid protein was detected using the rabbit anti-HEV-3 capsid protein polyclonal antibody #2101 (diluted 1/5,000 in 5% horse serum) by incubation overnight at 4 °C. The generation of

this rabbit antiserum has been described previously [8]. The HCV NS5A protein was stained by incubation with the monoclonal antibody 9E10 [9] (purchased from Cell essentials) in a concentration of 0.44 µg/mL in PBS containing 5% horse serum for 1 hour at room temperature. After incubation with the primary antibody, cells were washed thrice with PBS prior to incubation with the corresponding secondary antibodies labelled with Alexa-Flour 488 and 555 (Invitrogen, 2 µg/mL in 5% horse serum in PBS) for 1–2 hours at room temperature protected from light. Cells were washed two times with PBS before cellular DNA was stained by a short incubation at room temperature with 4′, 6-diamidino-2-phenylindole (DAPI, Invitrogen, 1 µg/mL). Finally, three washes with water were performed prior to imaging. Microscopic images were taken using the Keyence BZX800 microscope (4×, 10×, 20× objectives).

RNA in situ hybridization assay For binding and entry assays, HepG2/C3A, MLT, Hep56.1D and AML12 ($6x10^4$/well) cells were seeded into 18-well ibidi plates (ibidi GmbH) the day before inoculation. Cells were inoculated with HEV (MOI=3) for 2 h at 4 °C (binding) or for 1 h, 4 h or 8 h at 37 °C (entry kinetics). The inoculum was removed, cells were washed in PBS and fixed in 4% PFA for 15 min. To test viral RNA trafficking to the endolysosomal compartments, cells were inoculated for 2 h at 37 °C, followed by a medium change and additional 6 h of incubation. Cells were permeabilized in 0.2% Triton-X100. For detection of the ORF2 capsid protein, immunostaining with HEV ORF2 capsid protein was performed using the Alexa Fluor TM 488 Tyramide SuperBoostTM Kit (Thermo Scientific) according to the manufacturer's protocol, together with the rabbit anti-HEV-3 ORF2 capsid protein polyclonal antibody diluted (1:5,000) in blocking solution by incubation overnight at 4 °C. For the co-detection with HEV RNA, RNAscope® Fluorescent Multiplex Kit version 2 (ACDBio) was used according to the manufacturer's protocol. The positive strand HEV RNA was targeted by the specific ORF1 probe (#579831). Human LAMP1 was stained using LAMP1 Antibody (H4A3) (sc-20011, Santa Cruz, 0.5 µg/ml) and murine LAMP2 using anti-LAMP2 antibody (GL2A7) (ab13524, Abcam, 1 µg/ml). The cells were mounted in ibidi Mounting Medium with DAPI (ibidi GmbH). Multichannel z-series with a z-spacing of 0.28 µm were acquired using a Leica SP8 confocal microscope. A 63-oil immersion objective was used for all images. Five to seven frames per condition were imaged. Maximum projections of full z-series are shown. Images were processed using ImageJ. CellProfiler was

used for the quantification of HEV genomes per cell and association analysis of HEV genome and capsid protein.

Cathepsin L cleavage assay

Recombinant murine cathepsin L (1515-CY-010, R&D Systems) was activated for 1 h at RT in activation buffer (50 mM sodiumcitrat, 150 mM NaCl, 1 mM EDTA, 0.615% CHAPS detergent, pH 3). Purified recombinant ORF2 capsid protein (4 µg, kindly provided by Thomas Krey and George Ssebyatika, Institute of Biochemistry, Universität zu Lübeck) was incubated with or without recombinant murine cathepsin L (10 µg/mL) in reaction buffer (100 mM sodium acetate, 1 mM EDTA, 5 mM DTT, pH 5.5) for 16 hours at 25 °C. As a cathepsin cleavage control, 1 µM K11777 (MedChemExpress) or DMSO as a solvent control was included in the reaction mixture. Samples were then mixed with 4× Laemmli buffer (BioRad), heated to 95 °C for 5 minutes, and subjected to sodium dodecyl sulfate-polyacrylamide gel electrophoresis (SDS-PAGE) followed by Coomassie blue staining. The destained SDS-PAGE was imaged using an Odyssey Imager (LI-COR Biotech GmbH).

Flow cytometry

ISRE-dependent GFP expression was validated in addition to microscopy by flow cytometry. For this, ISRE reporter cell lines were electroporated with *in vitro* transcribed full-length HEV RNA or tRNA. After 3 days of incubation, cells were washed once with PBS and after incubation with trypsin diluted in DMEM medium and transferred into a 1.5 mL tube. Cells were sedimented by centrifugation at 200 x g for 5 min at 4 °C and the pellet resuspended in 3% PFA, followed by an incubation for 30 min at room temperature. The PFA was washed away by two additional centrifugation steps and resuspension in flow cytometry buffer (2% FCS in PBS). Samples were transferred to a v-shaped-bottom 96-well plate and GFP expression quantified using the CytoFLEX LX (Beckman Coulter).

Real-time quantitative PCR

Quantification of ISG expression was done from total RNA extracted using the NucleoSpin RNA kit (Macherey-Nagel) according to manufacturer's instructions. Extracted RNA was used for cDNA synthesis using the TaKaRa PrimeScript RT Master Mix (TaKaRa Bio) according to the manufacturer's instructions. Real-time quantitative PCR was performed on a LightCycler480 (Roche) using the GoTaq

2-step RT-qPCR Kit (Promega) according to the manufacturer's instructions. Primer sequences for amplification of each gene product are listed in Table 1. Glyceraldehyde 3-phosphate dehydrogenase (GAPDH) mRNA expression levels were used as internal reference, when relative gene expression was calculated using the 2-ddCT method [10].

Table 1: Primer

hISG15	sense	5'-CGCAGATCACCCAGAAGATCG-3'
	antisense	5'-TTCGTCGCATTTGTCCACCA-3'
hMX1	sense	5'-GTTTCCGAAGTGGACATCGCA-3'
	antisense	5'-CTGCACAGGTTGTTCTCAGC-3'
hGAPDH	sense	5'-GAAGGTGAAGGTCGGAGTC-3'
	antisense	5'-GAAGATGGTGATGGGATTTC-3'
hIFIT1 [11]	sense	5'- GGAATACACAACCTACTAGCC-3'
	antisense	5'- CCAGGTCACCAGACTCCTCA-3'
hIFNb [12]	sense	5'-AGGACAGGATGAACTTTGAC-3'
	antisense	5'-TGATAGACATTAGCCAGGAG-3'
mIsg15 [13]	sense	5'-GGTGTCCGTGACTAACTCCAT-3'
	antisense	5'-TGGAAAGGGTAAGACCGTCCT-3'
mMx1 [14]	sense	5'-GACCATAGGGGTCTTGACCAA-3'
	antisense	5'-AGACTTGCTCTTTCTGAAAAGCC-3'
mGapdh [13]	sense	5'-CCCACTAACATCAAATGGGG-3'
	antisense	5'-CCTTCCACAATGCCAAAGTT-3'
mIfit1 [14]	sense	5'-CTGAGATGTCACTTCACATGGAA-3'
	antisense	5'-GTGCATCCCCAATGGGTTCT-3'
mIfnb [14]	sense	5'-CTGCGTTCCTGCTGTGCTTCTCCA-3'
	antisense	5'-TTCTCCGTCATCTCCATAGGGATC-3'

RNA sequencing and transcriptome analyses

For analyzing gene expression patterns by RNA-seq, cells were lysed according to manufacturer's instructions of the NucleoSpin RNA kit (Macherey-Nagel). Cell lysates were thawed after storage at -80 °C at room temperature and passed ten times through a narrow-bore syringe (Omican-F, 1 mL, 0.3 × 12 mm2, 9161502, B Braun) prior to total RNA extraction according to the manufacturer's instructions. Quality and quantity of extracted RNA were analyzed using the NanoDrop One (Thermo Scientific) and the 4150 TapeStation system using the RNA ScreenTape reagents (Agilent). Library preparation was performed using the NEBNext UltraExpress RNA Library Prep Kit (New England Biolabs) and the recommended related reagents by following the manufacturer's instructions. The library was sequenced with P2 reagents (Illumina) on the Illumina NextSeq 1000 sequencing platform. RNA-seq data of

PHH,were used from previous publications [15,16] and are available at NCBI GEO database (GSE274780) or on request.

Raw FASTQ sequencing reads were checked for quality using FastQC (version 0.12.1; available online at: http://www.bioinformatics.babraham.ac.uk/projects/fastqc), followed by quality trimming with Trimmomatic [17]. Low-quality and overrepresented sequences were trimmed. The trimmed reads from PMH, MLT, and 56D cells were mapped to the Mus musculus (GRCm39) reference genome (GCF_000001635.27), while the trimmed reads from HepG2 cells were mapped to the Homo sapiens (GRCh38) reference genome (GCF_000001405.40), both obtained from National Center for Biotechnology Information (NCBI). Mapping was performed using HISAT2 version 2.2.1 [18]. Picard was used to remove optical duplicates (available online at: https://broadinstitute.github.io/picard/). FeatureCounts version 2.0.6. [19] was used to generate gene counts, determining how many reads mapped to each gene in the reference genome. Unmapped reads from HISAT2 were mapped to the viral genome using Burrows-Wheeler Aligner version 0.7.18 [20], and were used to algin to the HEV strain Kernow-C1 (GenBank accession Nr. HQ709170.1). Samtools version 1.21 [21] was used to sort, index, and calculate coverage. Viral coverage plots were visualized in R using the ggplot2 library.

Visualization was performed in R (version 4.5.1), using libraries for data sorting, including dplyr [22], and tidyr [23]. Differential gene expression analysis for PMH datawas done using edgeR [24], while DESeq2 [25] was used for HepG2 , MLT, and 56D cell line-derived data. Heatmaps were generated with ComplexHeatmap [26], and volcano plots were created with EnhancedVolcano [27].

Statistical analysis

Graphical visualization of experimental data and statistical analyses were performed with GraphPad Prism 10.2.3 (GraphPad Software). Prior to statistical analyses, data were tested for normal and lognormal distribution. Based on the relative likelihood, data was transformed into logarithmic format if recommended. Statistical significance was calculated using either two-tailed, paired t-test, ordinary one-way or two-way ANOVA with either Sidak's or Dunnett's multiple comparisons correction as indicated in the respective figure legend. Significance levels were visualized as follows: not significant p-value >0.05; * = p-value <0.05; ** = p-value <0.01; *** = p-value <0.001; **** = p-value <0.0001.

Data availability

All relevant data generated and analyzed during the study are included in the manuscript and supplementary files. The generated raw RNA-seq data were submitted to the NCBI GEO database and can be accessed under the accession number GSE319021.

Writing assistance

Throughout the manuscript preparation, the authors utilized artificial intelligence-based assistance offered by Ruhr University Bochum to improve readability and grammatical accuracy of the text. All suggested changes were carefully reviewed, verified and approved by the authors. The authors take full responsibility for the content and scientific accuracy of the publication.

References

[1] Frentzen A, Anggakusuma, Gürlevik E, et al. Cell entry, efficient RNA replication, and production of infectious hepatitis C virus progeny in mouse liver-derived cells. Hepatology. 2014;59:78–88.

[2] Nguyen HT, Shukla P, Torian U, et al. Hepatitis E virus genotype 1 infection of swine kidney cells in vitro is inhibited at multiple levels. J Virol. 2014;88:868–877.

[3] Schaller T, Appel N, Koutsoudakis G, et al. Analysis of hepatitis C virus superinfection exclusion by using novel fluorochrome gene-tagged viral genomes. J Virol. 2007;81:4591–4603.

[4] Froggatt HM, Harding AT, Chaparian RR, et al. ETV7 limits antiviral gene expression and control of influenza viruses. Sci Signal. 2021;14. DOI: 10.1126/scisignal.abe1194.

[5] Todt D, Friesland M, Moeller N, et al. Robust hepatitis E virus infection and transcriptional response in human hepatocytes. Proc Natl Acad Sci U S A. 2020;117:1731–1741.

[6] Meister TL, Klöhn M, Steinmann E, et al. A Cell Culture Model for Producing High Titer Hepatitis E Virus Stocks. J Vis Exp. 2020. DOI: 10.3791/61373.

[7] Guo H, Xu J, Situ J, et al. Cell binding tropism of rat hepatitis E virus is a pivotal determinant of its zoonotic transmission to humans. Proc Natl Acad Sci U S A. 2024;121:e2416255121.

[8] Behrendt P, Friesland M, Wißmann J-E, et al. Hepatitis E virus is highly resistant to alcohol-based disinfectants. J Hepatol. 2022;76:1062–1069.

[9] Lindenbach BD, Evans MJ, Syder AJ, et al. Complete replication of hepatitis C virus in cell culture. Science. 2005;309:623–626.

[10] Livak KJ, Schmittgen TD. Analysis of relative gene expression data using real-time quantitative PCR and the 2(-Delta Delta C(T)) Method. Methods. 2001;25:402–408.

[11] Li D, Swaminathan S. Human IFIT proteins inhibit lytic replication of KSHV: A new feed-forward loop in the innate immune system. PLoS Pathog. 2019;15:e1007609.

[12] Colpitts CC, Ridewood S, Schneiderman B, et al. Hepatitis C virus exploits cyclophilin A to evade PKR. Elife. 2020;9. DOI: 10.7554/eLife.52237.

[13] Qian G, Zhang Y, Liu Y, et al. Glutamylation of an HIV-1 protein inhibits the immune response by hijacking STING. Cell Rep. 2023;42:112442.

[14] Zhang Y, Kinast V, Sheldon J, et al. Mouse Liver-Expressed Shiftless Is an Evolutionarily Conserved Antiviral Effector Restricting Human and Murine Hepaciviruses. Microbiol Spectr. 2023;11:e0128423.

[15] Klöhn M, Gömer A, He Q, et al. The glutamate receptor antagonist ifenprodil inhibits hepatitis E virus infection. Antimicrob Agents Chemother. 2024;68:e0103524.

[16] Kinast V, Andreica I, Ahrenstorf G, et al. Janus kinase-inhibition modulates hepatitis E virus infection. Antiviral Res. 2023;217:105690.

[17] Bolger AM, Lohse M, Usadel B. Trimmomatic: a flexible trimmer for Illumina sequence data. Bioinformatics. 2014;30:2114–2120.
[18] Kim D, Paggi JM, Park C, et al. Graph-based genome alignment and genotyping with HISAT2 and HISAT-genotype. Nat Biotechnol. 2019;37:907–915.
[19] Liao Y, Smyth GK, Shi W. featureCounts: an efficient general purpose program for assigning sequence reads to genomic features. Bioinformatics. 2014;30:923–930.
[20] Li H. Aligning sequence reads, clone sequences and assembly contigs with BWA-MEM. [place unknown]; 2013.
[21] Danecek P, Bonfield JK, Liddle J, et al. Twelve years of SAMtools and BCFtools. Gigascience. 2021;10. DOI: 10.1093/gigascience/giab008.
[22] Wickham H, François R, Henry L, et al. CRAN: Contributed Packages. [place unknown]: [publisher unknown]; 2014.
[23] Wickham H, Vaughan D, Girlich M. CRAN: Contributed Packages. [place unknown]: [publisher unknown]; 2014.
[24] Chen Y, Chen L, Lun ATL, et al. edgeR v4: powerful differential analysis of sequencing data with expanded functionality and improved support for small counts and larger datasets. Nucleic Acids Res. 2025;53. DOI: 10.1093/nar/gkaf018.
[25] Love MI, Huber W, Anders S. Moderated estimation of fold change and dispersion for RNA-seq data with DESeq2. Genome Biol. 2014;15:550.
[26] Gu Z, Eils R, Schlesner M. Complex heatmaps reveal patterns and correlations in multidimensional genomic data. Bioinformatics. 2016;32:2847–2849.
[27] Kevin Blighe. EnhancedVolcano. [place unknown]: Bioconductor; 2018.

Supplementary figures

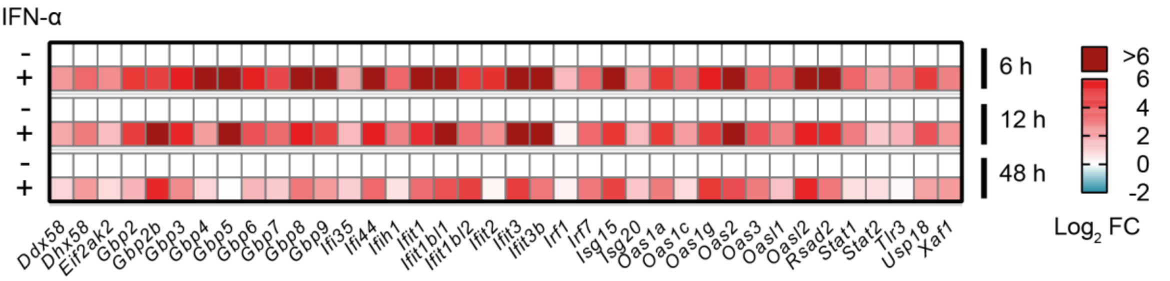

Figure S1: Upregulation of interferon-stimulated gene expression in primary mouse hepatocytes upon murine interferon-α treatment. Primary mouse hepatocytes from one CD-1 donor mice were treated with murine interferon-α [100 IU/ml] for 6, 12 and 48 hours. Fold change (FC) in mRNA levels of genes characterized under the term "interferon-stimulated genes" of treated and untreated hepatocytes were measured by total RNA-sequencing.

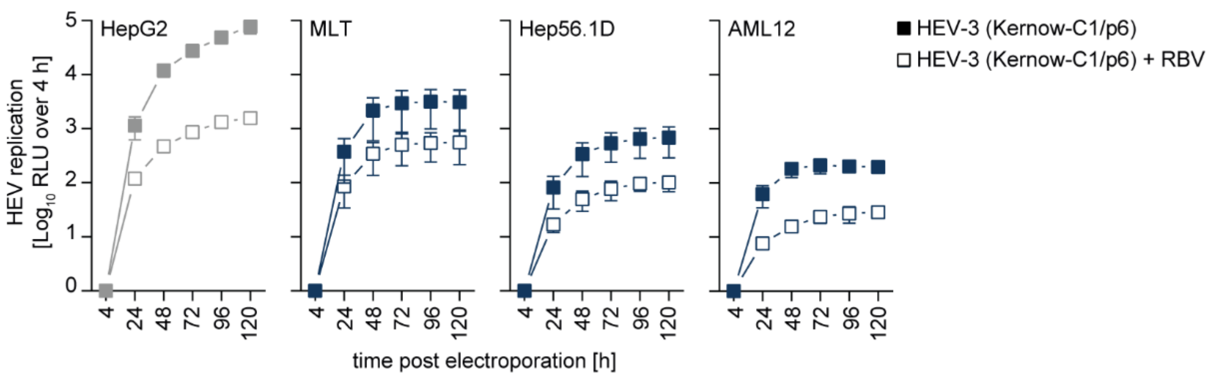

Figure S2: Long-term HEV replication dynamics in murine and human liver cell lines. Replication fitness of HEV-3 (Kernow-C1/p6) subgenomic replicon in human HepG2 and murine MLT, Hep56.1D and AML12 cell lines with and without Ribavirin (RBV, 50 µM) treatment. Replication was measured at indicated time points in correlation to Gaussia luciferase activity. The presented data are relative light units (RLU) normalized to 4 hours post electroporation. Mean values ± SD from n = 3 experiments are displayed.

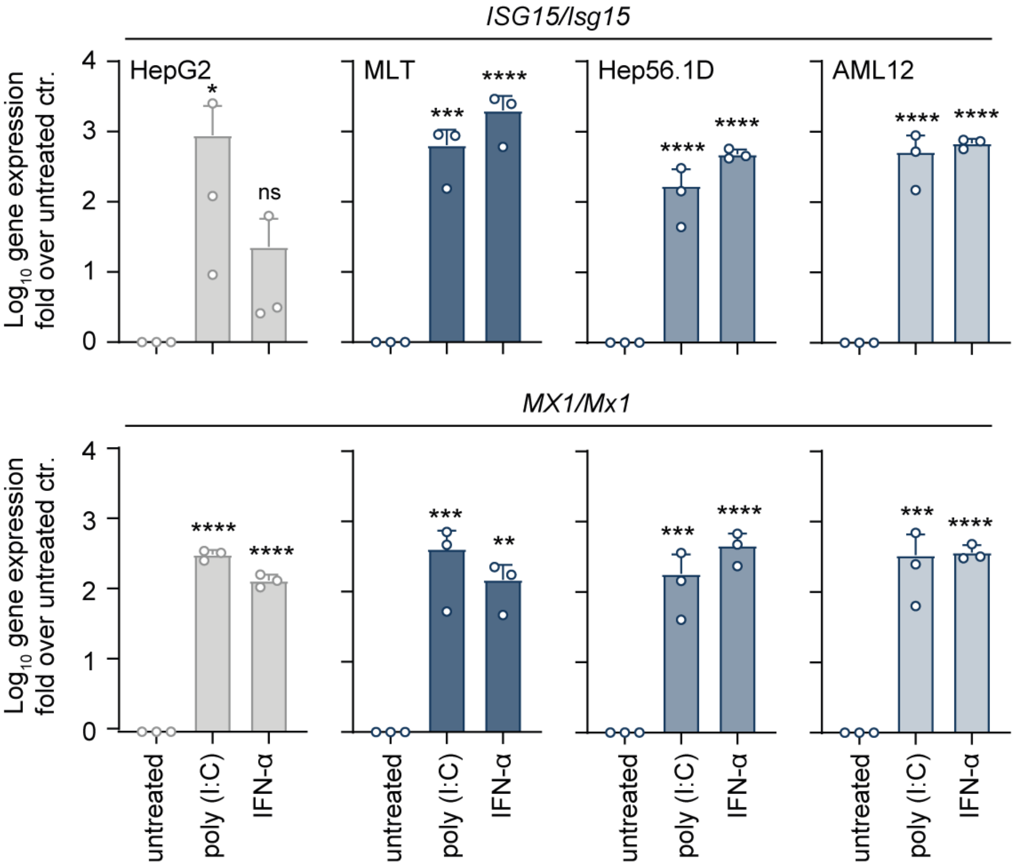

Figure S3: Characterization of human and murine antiviral responses to non-HEV-related immune stimuli. Human HepG2 and murine MLT, Hep56.1D and AML12 cells were either untreated, transfected with 0.5 µg poly(I:C) or treated with species-specific interferon-α (IFN-α, 1,000 IU/ml). After 16 h of incubation, cells were lysed and antiviral gene expression quantified. Statistical significance was calculated on log transformed data using an ordinary one-way ANOVA with Dunnett multiple comparison correction. N.s., not significant with p-value >0.05; * = p-value <0.05; ** = p-value <0.01; **** = p-value <0.0001.

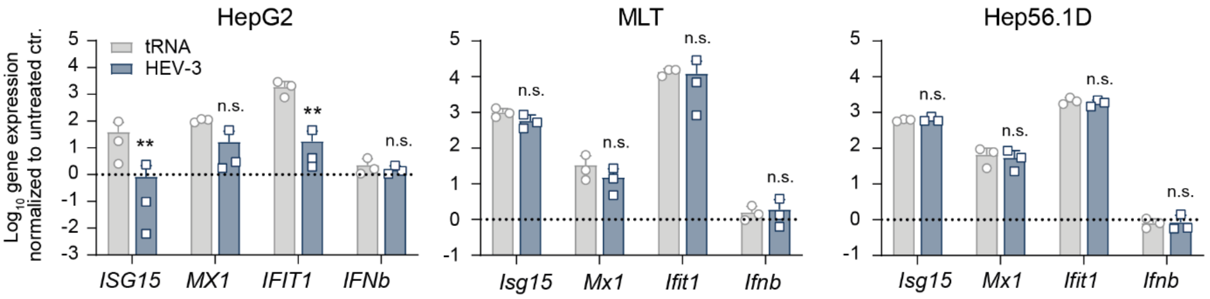

Figure S4: Interferon-stimulated gene expression upon interferon treatment is reduced in HEV-3 transfected human but not murine hepatic cells. Expression of representative interferon-stimulated genes upon interferon-α treatment [100 IU/ml] of HEV-3 full length RNA or tRNA-transfected human HepG2 and murine MLT and Hep56.1D cells. Mean and individual values + SD from n = 3 experiments are shown. Statistical significance was calculated on log transformed data using an ordinary two-way ANOVA with Šídák's multiple comparison correction. N.s., not significant with p-value >0.05; ** = p-value <0.01.

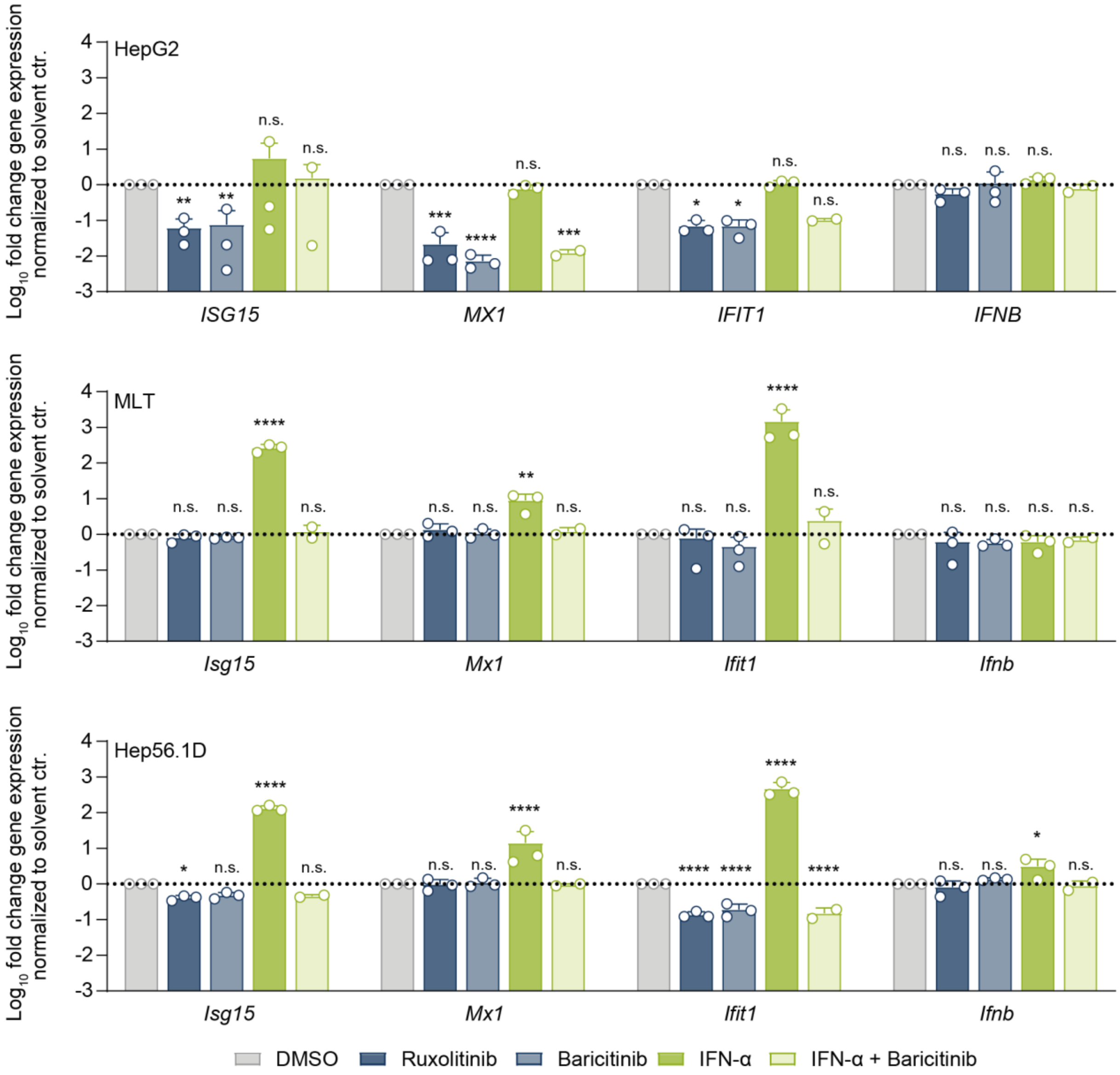

Figure S5: Validation of Janus kinase inhibitor activity in human and murine liver cell lines. Expression of representative interferon-stimulated genes upon Janus kinase inhibitor treatment with either ruxolitinib or baricitinib [2 μM], IFN-α treatment [100 IU/ml] or combinational treatment of HEV-3 subgenomic replicon-transfected human HepG2 cells and murine MLT and Hep56.1D cells. The data presented were normalized to DMSO-treated cells as solvent control (ctr.). Mean and individual values + SD from n = 2-3 experiments are shown. Statistical significance was calculated on log transformed data using an ordinary two-way ANOVA with Dunnett multiple comparison correction. N.s., not significant with p-value >0.05; * = p-value <0.05; ** = p-value <0.01; **** = p-value <0.0001.

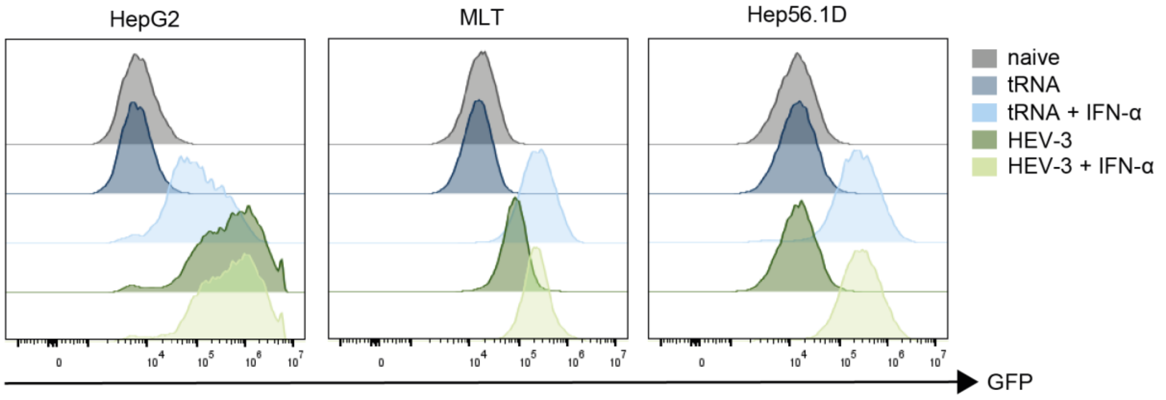

Figure S6: ISRE activity upon HEV-3 replication. Flow cytometry-based ISRE-dependent GFP expression in human HepG2 and murine MLT and Hep56.1D cells transfected with either tRNA or *in vitro* transcribed HEV-3 full-length RNA. Treatment with IFN-α [100 IU/ml] for 16 hours prior to cell fixation served as a positive control. Representative analyses of n=3 experiments are shown.

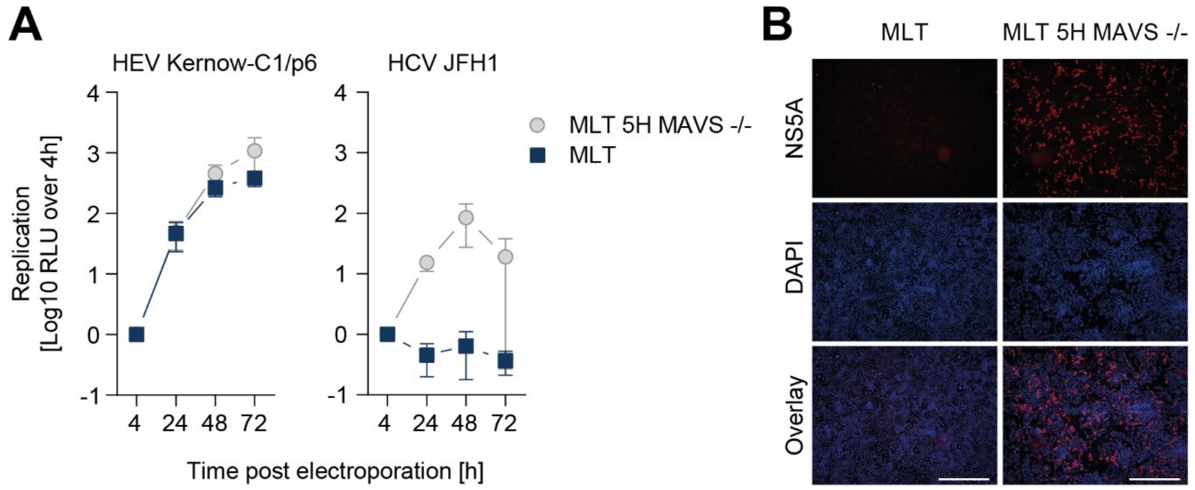

Figure S7: MAVS knockout facilitates HCV but not HEV replication. (A) Replication fitness of HEV-3 (Kernow-C1/p6, left) and HCV (JFH1, right) subgenomic replicons in murine liver cell lines with and without MAVS expression. Replication was measured at indicated time points in correlation to luciferase activity. The presented data are relative light units (RLU) normalized to 4 hours post electroporation. Mean values ± SD from n = 4 experiments are displayed. (B) Representative images of NS5A expression (red) of HCV (JFH1) sub genomic replicon harboring MLT cells with and without MAVS knockout. DAPI=blue; scale bar= 1000 µm.

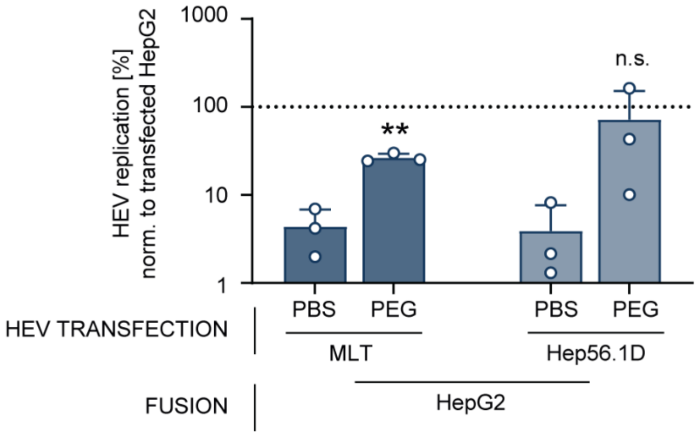

Figure S8: Fusion with human HepG2 cells enhances HEV-3 replication in murine hepatic cell lines. Murine MLT and Hep56.1D cells were transfected with a HEV-3 subgenomic reporter replicon prior to polyethylene glycol-mediated fusion to HepG2 cells. As unfused control, co cultures of murine and human cells were incubated with PBS. HEV replication was quantified by luciferase readout at 24 hours post fusion. Replication levels in HEV-transfected HepG2 cells co-cultivated with naïve HepG2 cells were used for normalization (norm.). Mean and individual values + SD from n = 3 experiments are shown. Statistical significance was calculated using unpaired multiple t-test with Holm-Šídák method for multiple comparison correction. N.s., not significant with p-value >0.05; * = p-value <0.05; ** = p-value <0.01.

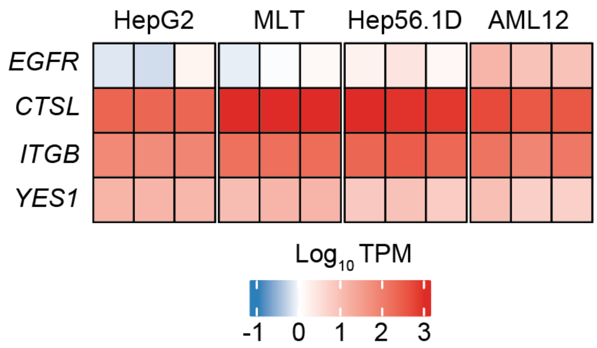

Figure S9: Expression of HEV entry factors in human and murine hepatic cell lines. Human HepG2 and murine MLT, Hep56.1D and AML12 cells were lysed and subjected to total RNA-sequencing. Transcripts per million (TPM) values from three individual passages of cells are shown.

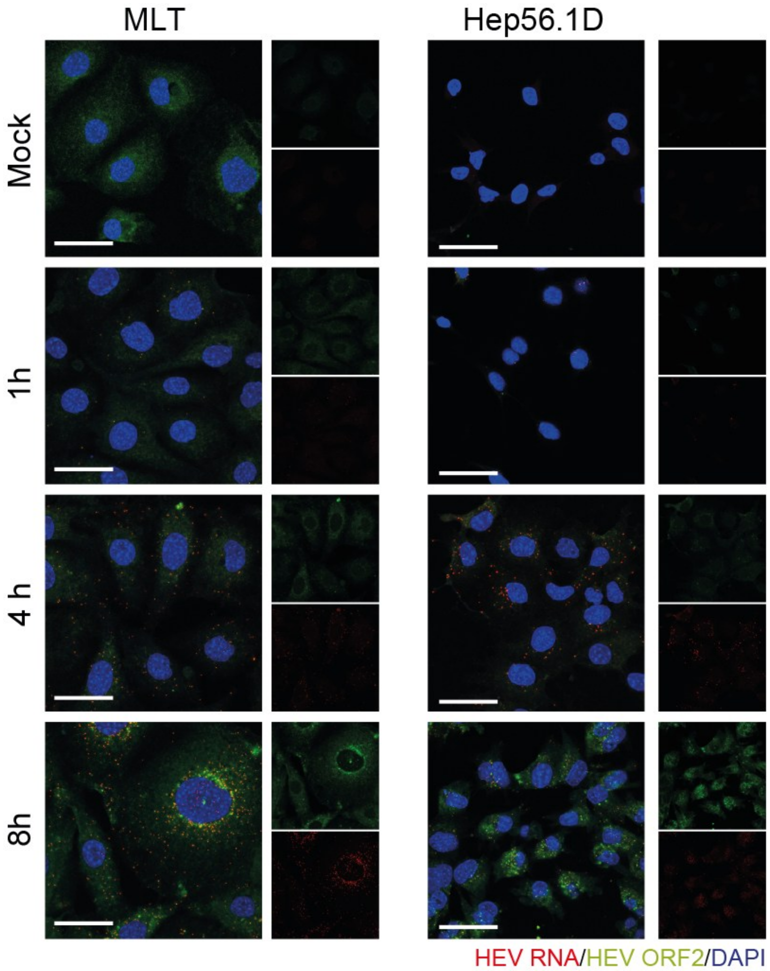

Figure S10: Detection of HEV RNA and HEV ORF2 capsid protein during internalization of naked HEV particles in murine liver cell lines. Representative images of HEV ORF2 capsid protein immunofluorescence staining and RNA in situ hybridization assay for detection of HEV RNA over the indicated time frame of n=3 are shown. HEV RNA=red; HEV ORF2 capsid protein=green, DAPI=blue; scale bar=50 µm.

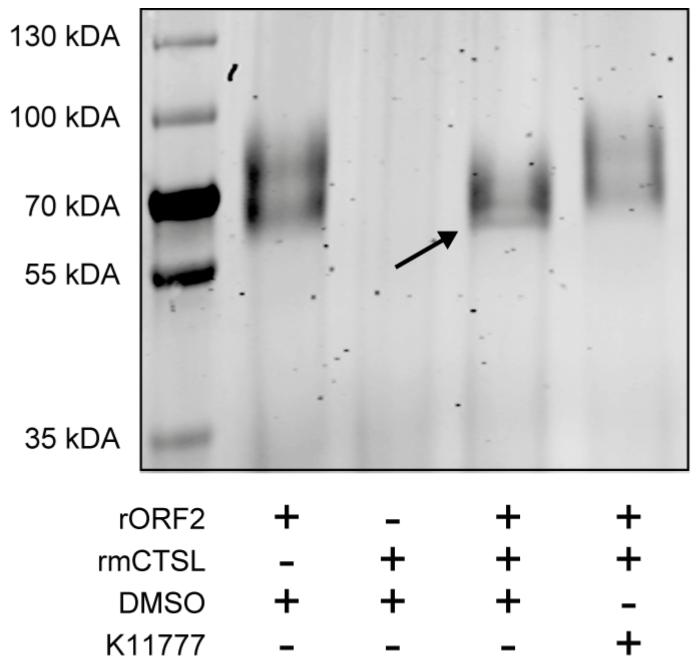

Figure S11: Murine CTSL cleaves ORF2 capsid protein *in vitro*. Recombinant ORF2 capsid protein (rORF2) was incubated with recombinant murine cathepsin L (rmCTSL). As a control for the cleavage reaction, reaction mixtures were supplemented with the cathepsin L inhibitor K11777 or DMSO as a solvent control. After 16 hour incubation, samples were analyzed by SDS-PAGE and Coomassie blue staining. The smaller-sized cleavage product is highlighted with an arrow.

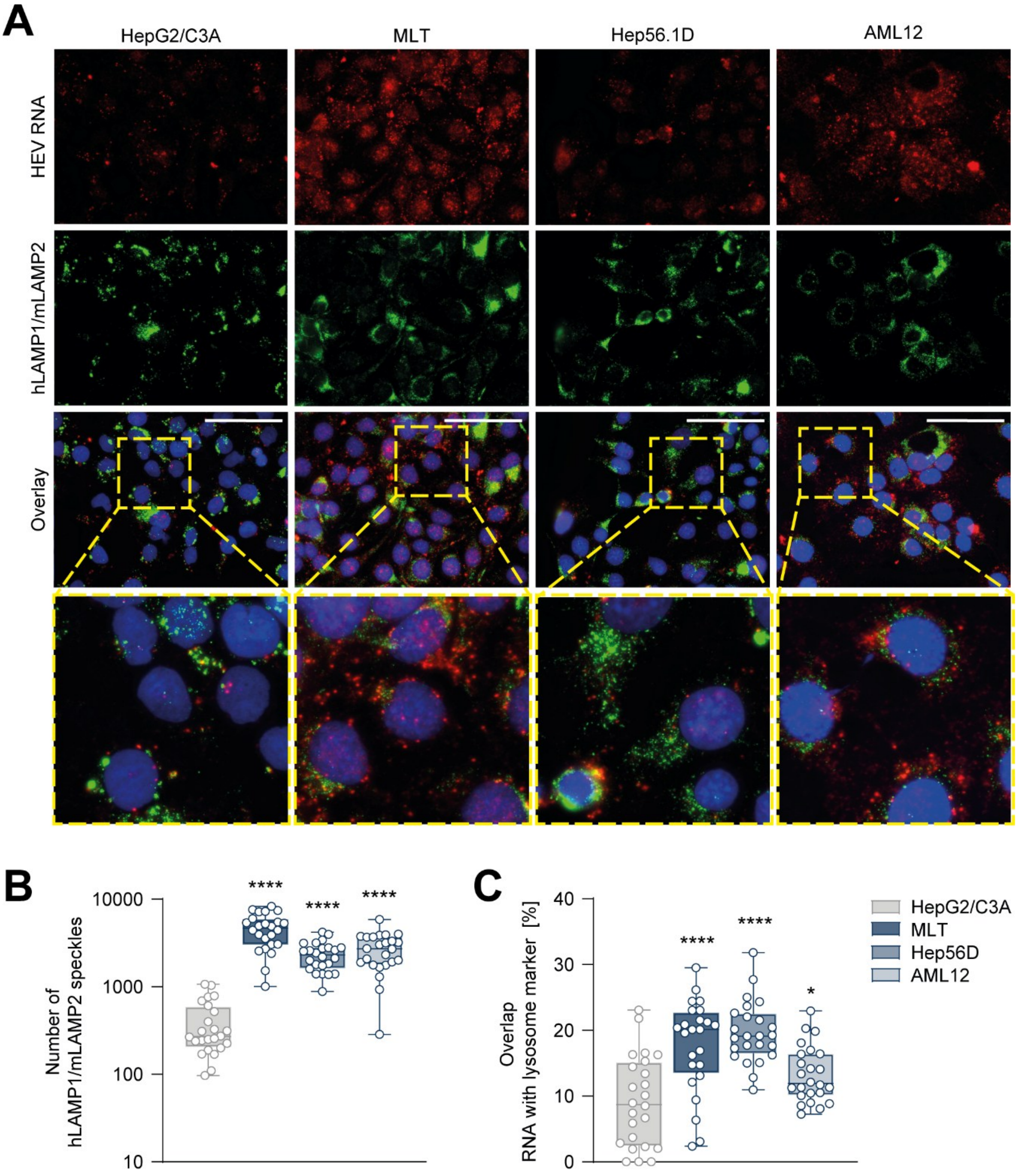

Figure S12: Increased HEV RNA and lysosome signal overlap in murine liver cells. (A) Representative images of lysosomal marker immunofluorescence staining and RNA in situ hybridization assay for detection of HEV RNA at 8 hours post inoculation. HEV RNA=red; human (h)LAMP1/murine (m)LAMP2=green, DAPI=blue; scale bar=80 µm. (B) Quantification of hLAMP1 and mLAMP2 speckles from panel A (C) Quantification of RNA and lysosome marker signal overlap from panel A. Eight frames per biological replicate (n=3 in total) were analyzed per cell line. Individual values per analyzed frame are shown. Statistical significance was calculated on single values per cell line using ordinary one-way ANOVA with Dunnett multiple comparison correction. N.s., not significant with p-value >0.05; * = p-value <0.05; ** = p-value <0.01, *** = p-value <0.001, **** = p-value <0.0001.
